# MTHFR inhibits TRC8‐mediated HMOX1 ubiquitination and regulates ferroptosis in ovarian cancer

**DOI:** 10.1002/ctm2.1013

**Published:** 2022-09-23

**Authors:** Xiang Wang, Zhijie Xu, Xinxin Ren, Xi Chen, Qiaoli Yi, Shuangshuang Zeng, Abhimanyu Thakur, Zhicheng Gong, Yuanliang Yan

**Affiliations:** ^1^ Department of Pharmacy Xiangya Hospital, Central South University Changsha China; ^2^ Department of Clinical Pharmacy Affiliated Hangzhou First People's Hospital Zhejiang University School of Medicine Hangzhou China; ^3^ Department of Pathology Xiangya Hospital, Central South University Changsha China; ^4^ Institute for Rational and Safe Medication Practices National Clinical Research Center for Geriatric Disorders Xiangya Hospital, Central South University Changsha China; ^5^ Department of Pathology Zhejiang Provincial People's Hospital Hangzhou China; ^6^ Pritzker School of Molecular Engineering Ben May Department for Cancer Research University of Chicago Chicago Illinois USA

Dear Editor,

The study is the first to confirm that methylenetetrahydrofolate reductase (MTHFR) could inhibit HMOX1 ubiquitination degradation by competitive interaction with TRC8, followed by blocking the occurrence of ferroptosis in ovarian cancer (OV) cells, and promote the tumour cells growth. OV attributes to the world's second most familiar cause of gynaecologic cancer death.^1^ Recent research studies identified that the polymorphisms of MTHFR correlate with the risk of common gynaecological cancers.^2^, ^3^ However, there is a dearth of studies with in‐detail elucidation of functions and mechanisms of MTHFR in OV.

MTHFR is a key enzyme involved in folic acid metabolism.^2^ In this study, it was found that MTHFR was upregulated in OV cell lines (SKOV3, TOV112D, A2780 and OVCAR3) and tissues both in mRNA and protein levels (Figures 1A,B and S1A). The mRNA and protein expression of MTHFR was found to be higher in A2780 and OVCAR3 cells, which led us to the selection of A2780 and OVCAR3 cells for further study. The higher expression of MTHFR was associated with poor overall survival (OS), progression‐free survival (PFS) and post‐progression survival (Figure S1B–D). As shown in Figures 1C–F and S2A–C, it was found that the colony numbers and cell viability were obviously decreased in MTFHR‐interfering cells compared to the control cells, as determined by the colony formation and CCK8 assays, respectively. In addition, we applied a lentivirus‐medicated expression system to stably overexpress MTHFR in MTFHR‐interfered OV cells (Figures 1G and S2D). By colony formation and CCK8 assays, the overexpression of MTHFR significantly reversed the inhibition effects of MTHFR knock‐down in OV cells (Figures 1H–J and S2E,F). Cisplatin, cisplatinum or *cis*‐diamminedichloroplatinum(II) (CDDP) are a classical antitumour drug, which could be considered an inducer for ferroptosis.^4^, ^5^ Moreover, the expression of MTHFR in OV cells was decreased in a dose‐dependent manner of CDDP (Figure S3A,B). Interestingly, the concentration gradient‐based treatment with CDDP, MTHFR downregulation could inhibit the colony formation ability and viability in OV cells (Figure S3C–F). Additionally, an overexpressed level of MTHFR significantly reversed the effect of MTHFR knock‐down in MTHFR‐interfered OV cells under CDDP treatment (Figure S3G–J). Therefore, it is apparent to examine the relationship between MTHFR and drug sensitivity, and their contribution towards the therapeutics of OV.

**FIGURE 1:**
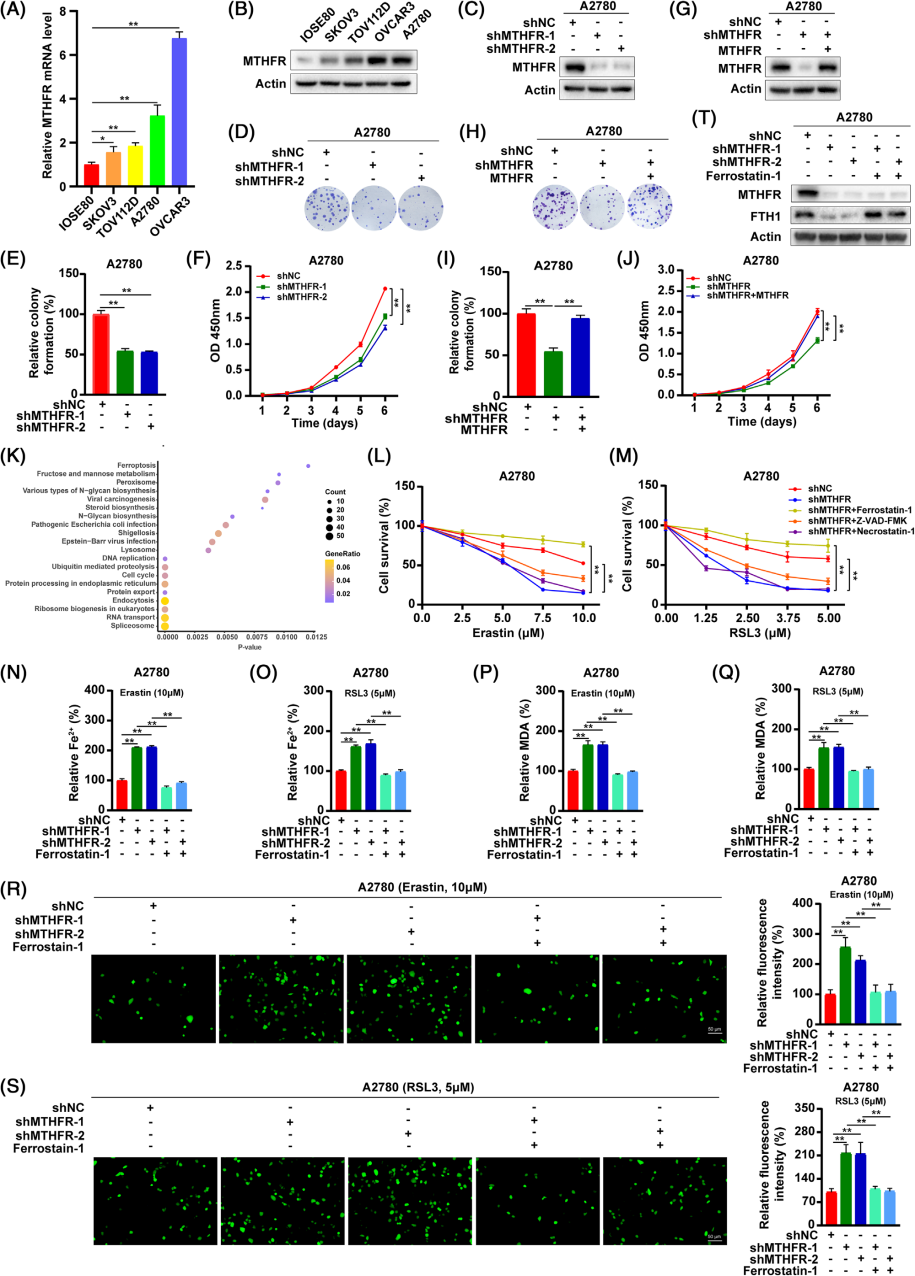
Methylenetetrahydrofolate reductase (MTHFR) promoted the colony formation and proliferation of OV cells and inhibited the ferroptosis in OV: (A) the mRNA expression of MTHFR in normal ovary cell line and OV cell lines; (B) the protein levels of MTHFR in normal ovary cell line and OV cell lines; (C) the western blot assay of MTHFR interfering in A2780 cells; (D and E) the colony formation assay was used to evaluate the effects of MTHFR knock‐down on the growth of A2780 cells; (F) the influences of MTHFR‐interference on the proliferation of A2780 cells; (G) overexpressed MTHFR in MTHFR knock‐down of A2780 cells experimented by western blot; (H and I) the colony formation assay of overexpressed MTHFR in MTHFR‐interference A2780 cells; (J) the effects of overexpression MTHFR on the proliferation of MTHFR‐interference A2780 cells; (K) KEGG pathways enriched by the differential expression proteins; (L and M) MTHFR‐interference accelerated A2780 cells death induced by erastin (10 μM) or RSL3 (5 μM). CCK8 assays were used to tested the proliferation of A2780 cells with or without the cell death inhibitor including ferrostatin‐1 (5 μM), Z‐VAD‐FMK (10 μM) and necrostatin‐1 (50 μM); (N)–(Q) the influences of MTFHR‐interference on the accumulation of Fe^2+^ and MDA under the treatment of erastin (10 μM) or RSL3 (5 μM) in A2780 cells; (R and S) the intracellular ROS levels were evaluated following the treatment of erastin (10 μM) or RSL3 (5 μM) in MTHFR‐interfered A2780 cells. Data shown represent mean ± SD (*n* = 3) and (T) MTHFR‐interfered A2780 cells were treated with ferrostatin‐1 (5 μM) and the protein level of FTH1 in MTHFR knock‐down of A2780 cells was experimented by western blot. **p < .01

As shown in Table S1, total 2040 proteins, obtained from mass spectrometry, were differentially expressed after MTHFR knock‐down compared to the control group, including HMOX1. The KEGG functional enrichment analyses suggested that differentially expressed proteins were significantly related to ferroptosis through the LinkInterpreter module (Figure 1K). Ferroptosis is an iron‐dependent regulated cell death induced by lipid peroxidation.^6^ In addition, the model based on ferroptosis related‐genes was contribute to predict the prognosis of OV patients and induction of ferroptosis could enhance the inhibitory effect of CDDP on OV cells.^7^, ^8^ To ascertain this result, we examined the viability of OV cells under the treatment of ferroptosis inducers, erastin and RSL3. As shown in Figures 1L,M and S4A,B, the knock‐down of MTHFR increased erastin‐ or RSL3‐induced growth suppression in OV cells and ferroptosis inhibitor (ferrostatin‐1) could significantly reverse the growth suppression of MTHFR knock‐down. To further validate this observation, the OV cells were treated with erastin or RSL3, which could increase the Fe^2+^, lipid peroxidation products (MDA) and ROS levels, whereas these effects could be reversed by ferrostatin‐1 in OV cells (Figure S4C–J). Moreover, the knock‐down of MTHFR increased the accumulation of Fe^2+^, MDA and ROS and decreased the protein level of FTH1 under erastin or RSL3 treatment and could be reversed by ferrostatin‐1 in OV cells (Figures 1N–T and S5A–I). The overexpression of MTHFR significantly reversed the promotion effects of MTHFR knock‐down on the accumulation of Fe^2+^ and MDA in OV cells (Figure S5J–Q). Anti‐folate and anti‐methionine strategies have been addressed extensively, whereas the clinical benefits of these approaches were inconclusive. This finding provided new ideas and rationality for clinical potential of MTHFR‐based targeting therapy of ovarian cancer.

HMOX1 is a rate‐limiting enzyme that degrade the haem into Fe^2+^.^9^ HMOX1 had a dual role in the ferroptosis regulation.^10^ This dual mechanism of HMOX1 was already present at early 1999.^11^, ^12^ Excessive activation of HMOX1 could induce ferroptosis in cancer cells.^13^ However, HMOX1 can degrade the haem into Fe2+.^9^ However, HMOX1 decreases the bioavailability of haem and plays an important anti‐oxidative role in cancer cells.^14^ In a renal ischaemia‐reperfusion model, the upregulation of HMOX1 could inhibit ferroptosis of renal tissues.^15^ In addition, knock‐down HMOX1 in hepatocellular carcinoma cells could promote ferroptosis of cells.^16^ Nishizawa et al. also demonstrated that HMOX1 acted as an inhibitor of ferroptosis.^17^ The generation of Fe^2+^ promotes the synthesis of ferritin, which inhibits the occurrence of ferroptosis.^10^ To identify the downstream of MTHFR, we overlapped the ferroptosis related‐genes and differentially expressed proteins after MTHFR knock‐down and found HMOX1 was significantly downregulated^18^ (Figure 2A). Consisting with the mass spectrometry, HMOX1 was found significantly reduced in MTHFR‐deficient OV cells (Figures 2B and S6A). Moreover, the overexpression of HMOX1 in MTHFR‐deficient cells could not rescue the downregulated expression of MTHFR by shRNA, inferring that MTHFR regulated the ferroptosis of OV cells through influencing the expression of HMOX1 (Figures 2C and S6B). Then, colony formation and CCK8 experiments showed that the overexpression of HMOX1 significantly rescued the inhibition effect of MTHFR knock‐down (Figures 2D–F and S6C–E). Moreover, treatment with CDDP downregulated MTHFR and HMOX1 expression in a dose‐dependent manner in OV cells (Figure S6F–G). In MTHFR‐deficient OV cells, the overexpression of HMOX1, significantly reversed the enhanced sensitivity to CDDP in OV cells by MTHFR knock‐down (Figure S6H–M). Moreover, the overexpression of HMOX1 could significantly rescue the induced ferroptosis and downregulation of FTH1 in MTHFR‐deficient OV cells (Figures 2G–M and S6N–V).

**FIGURE 2:**
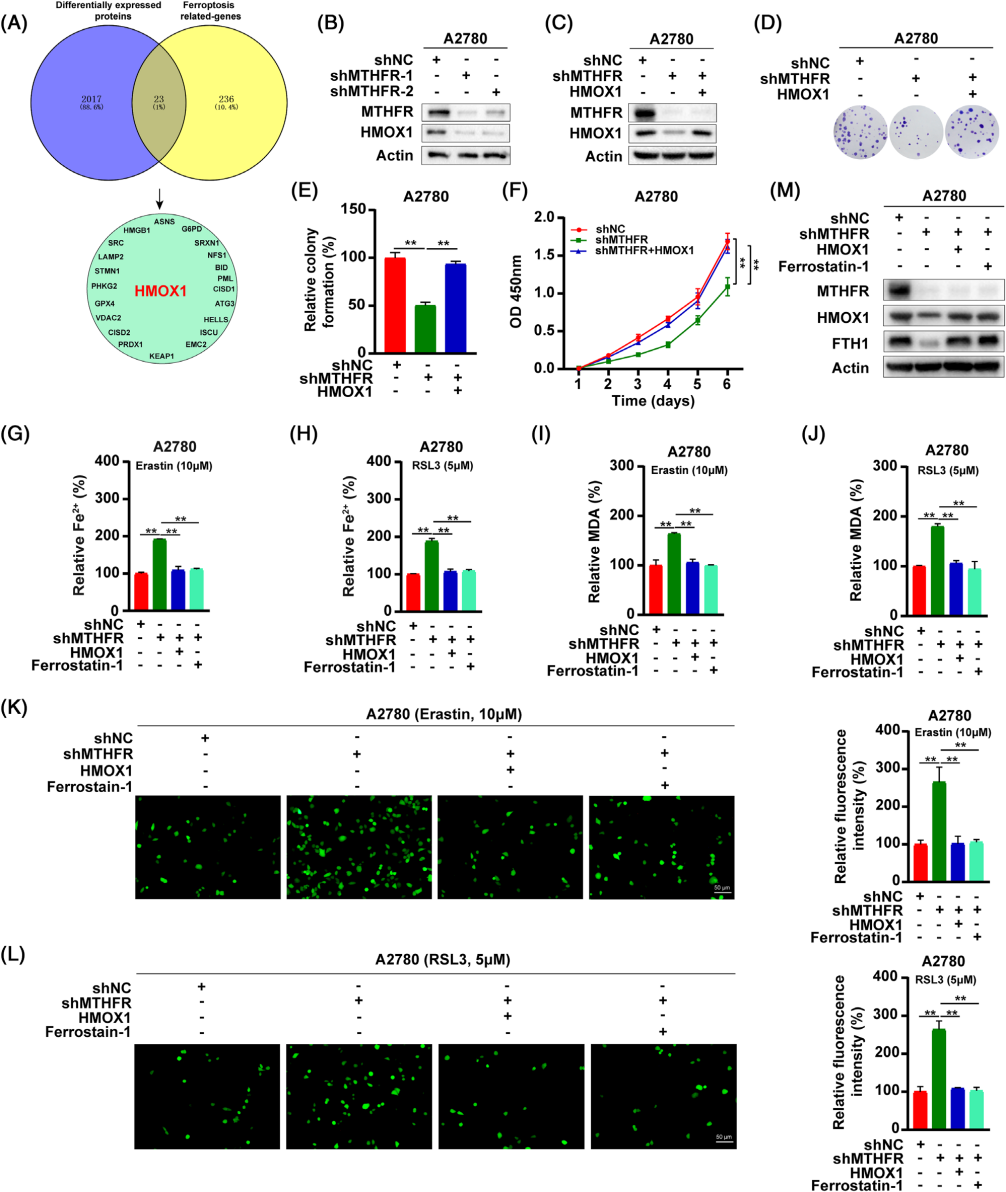
Methylenetetrahydrofolate reductase (MTHFR) regulated the ferroptosis of OV cells through influencing the expression of HMOX1: (A) the Venn diagram described the differentially expressed ferroptosis‐associated genes with the criteria of |foldchange| ≥ 1.5 in MTHFR‐interference A2780 cells; (B) western blot analysis detected the expression of HMOX1 in A2780 cells after knock‐down of MTHFR; (C) stable overexpressed HMOX1 in MTHFR knock‐down A2780 cells; (D)–(F) the colony formation and CCK8 assays were applied to test the effects of overexpressing HMOX1 on the growth and proliferation of MTHFR knock‐down A2780 cells; (G)–(L) the Fe^2+^, MDA and ROS levels in A2780 cells with the treatment of erastin (10 μM) or RSL3 (5 μM). Cells were treated by erastin (10 μM) or RSL3 (5 μM) with or without ferrostatin‐1 (5 μM) for 24 h. Then, Fe^2+^, MDA and ROS levels were experimented with the corresponding kit. Data shown represent mean ± SD (*n* = 3) and (M) stable overexpressed HMOX1 in MTHFR knock‐down A2780 cells and the MTHFR‐interfered cells were treated with ferrostatin‐1 (5 μM). The protein levels of HMOX1 and FTH1 in A2780 cells were experimented by western blot. **p < .01

Mechanically, nuclear factor erythroid 2 related factor 2 (NRF2) has been reported to regulate HMOX1 expression by transcriptional modification.^19^, ^20^ In renal carcinoma cells, HMOX1 was found to be degraded through ubiquitination by E3‐ligase TRC8, which resulted in the inhibition of cancer cell growth and migration.^21^ As MTHFR knock‐down did not influence HMOX1 transcription level (Figures 3A and S7A), as well as NRF2 expression (Figure S7B,C), it is apparent that MTHFR mediated HMOX1 upregulation might be through post‐translational modification. The cycloheximide chase assay revealed MTHFR knock‐down significantly decreased protein stability of HMOX1 in A2780 and OVCAR3 cells (Figures 3B,C and S7D). Furthermore, the knock‐down of MTHFR, increased ubiquitination especially K48‐linked but not K63‐linked polyubiquitination of HMOX1, whereas the overexpression of MTHFR could reverse this induction in MTHFR‐deficient OV cells (Figures 3D–G and S7E–K). In addition, Co‐IP showed MTHFR could form a complex both with HMOX1 and TRC8 in OV cells (Figures 3H and S7L). Interfered TRC8 by siRNA could abolish the increased ubiquitination of HMOX1 by MTHFR knock‐down in OV cells (Figures 3I and S7M). The knock‐down of MTHFR promoted the combination of TRC8 and HMOX1 (Figures 3J and S7N), whereas the overexpression of MTHFR reduced this integration in MTHFR‐deficient OV cells (Figures 3K and S7O).

**FIGURE 3:**
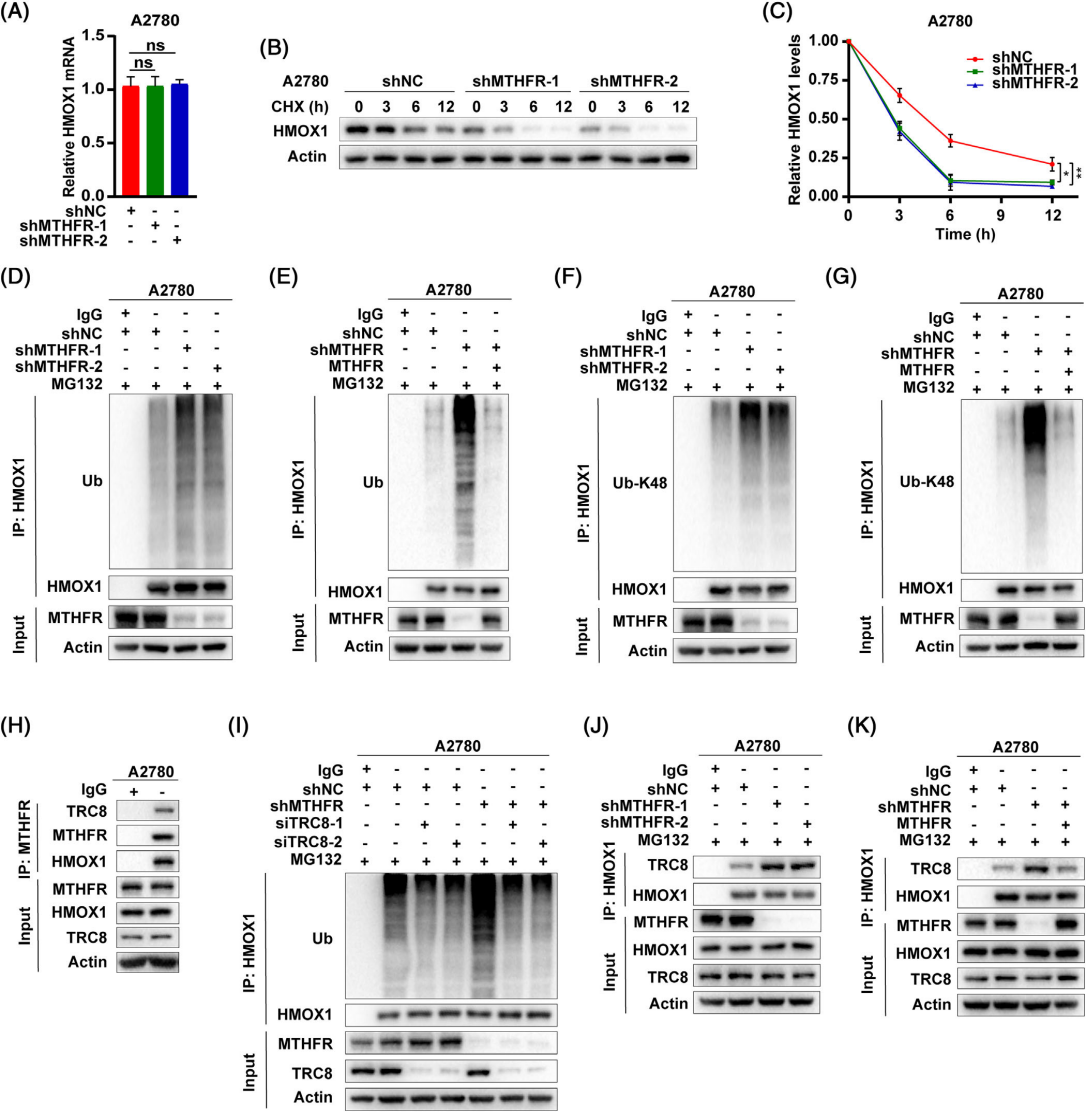
Methylenetetrahydrofolate reductase (MTHFR) stabilized HMOX1 by inhibiting K48‐linked ubiquitination of HMOX1 in OV cells: (A) the A2780 cells stably interfered with MTHFR and control, the RT‐PCR was used to detect the mRNA expression of HMOX1; (B and C) A2780 cells stably expressing MTHFR shRNAs or control were dealt with cycloheximide (CHX) (1 μM) and harvested at 0, 3, 6, 12 h. Then cell lysates were blotted with the indicated antibodies. Quantification of the MTHFR protein levels relative to actin; (D)–(G) A2780 cells were treated with MG132 (10 μM) overnight in indicated groups. Cell lysates were then subjected to immunoprecipitation with protein A/G agarose beads and blotted with the corresponding antibodies. The K48‐linked ubiquitination of HMOX1 was determined by western blot; (H) the binding of TRC8 and MTHFR with HMOX1 in A2780 cells assayed by Co‐IP; (I) the influence of si‐TRC8 on HMOX1 ubiquitination level in MTFHR knock‐down A2780 cells under the treatment of MG132 (10 μM) and (J and K) the effects of MTHFR on the combination of TRC8 and HMOX1 in A2780 cells under the treatment of MG132 (10 μM). Data shown represent mean ± SD (*n* = 3). *p < .05, **p < .01

Using tissue microarray, high levels of MTHFR and HMOX1 were observed in OV patients, which was correlated with the poor prognosis (Figures 4A,B and S8A–C). Moreover, pathology grade, Ki67 and EGFR were found to be positively correlated with MTHFR in OV patients significantly (Table 1). Additionally, positive correlation was found between MTHFR and HMOX1 expression in OV patients (Figure 4C,D). The OS and PFS of double positive OV patients, harboured both higher expression of MTHFR and HMOX1, were significantly poorer than that of double negative OV patients (Figure 4E,F). Moreover, xenograft tumour model showed that MTHFR downregulation apparently delayed tumour weights and volumes, whereas this effect could be rescued by HMOX1 overexpression (Figure 4G–I). The expression of MTHFR, HMOX1 and FTH1 in tissues was confirmed by IHC (Figure 4J).

**FIGURE 4:**
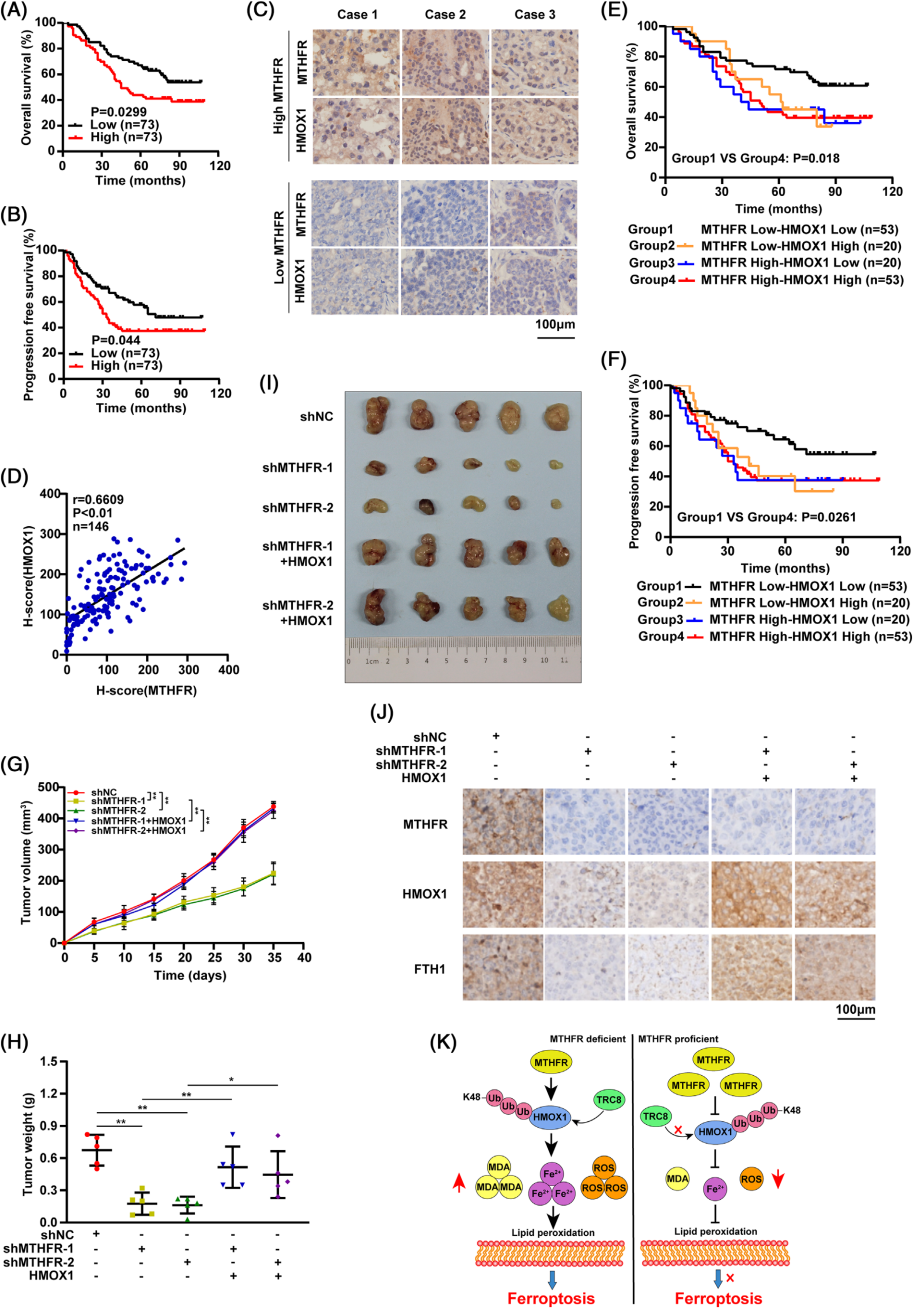
Methylenetetrahydrofolate reductase (MTHFR) expression was positively correlated with HMOX1 and poor prognosis in OV patients: (A and B) the relationship between MTHFR expression and overall survival (OS) or progression‐free survival (PFS), analysed by the OV tissue array; (C) the representative IHC images were shown; (D) the relationship between MTHFR and HMOX1 in 146 OV patients; (E and F) the association of MTHFR/HMOX1 expression with OS and PFS of OV patients was analysed. The expression of MTHFR and HMOX1 in OV patients was tested in the tissue array and scored. The patients were separated into four groups as mentioned, and the survival among the four groups was examined; (G)–(I) about 5 × 10^6^ of A2780 cells stable expressed shMTHFR, HMOX1 and control were injected into the 4‐week‐old female nude mice. The tumour weight (G) and tumour volume (H) of these five groups were statistically tested at 40 days after injection. The representative image of tumours from these five groups (I); (J) the results of OV tissues experimented by IHC. Data shown represent mean ± SD (*n* = 3) and (K) the diagram of the study. *p < .05, **p < .01

**TABLE 1:** The correlation between methylenetetrahydrofolate reductase (MTHFR) protein level and clinical features of OV patients

		MTHFR expression			
Characteristic	Total	Low (*n* = 53)	High (*n* = 54)	*χ* ^2^	*P*
**Age (years)**				.010	.919
≥50	51	25 (49%)	26 (51%)		
<50	56	28 (50%)	28 (50%)		
**Pathology grade**				4.457	**.035** ^*^
I–II	18	13 (72%)	5 (28%)		
III	89	40 (45%)	49 (55%)		
**Tumour size (cm)**				.895	.344
≥10	70	37 (53%)	33 (47%)		
<10	37	16 (43%)	21 (57%)		
**T**				5.616	**.018** ^*^
T1–T2	24	17 (71%)	7 (29%)		
T3	83	36 (43%)	47 (57%)		
**N**				.019	.890
N0	72	36 (50%)	36 (50%)		
N1	35	17 (50%)	18 (50%)		
**M**				.641	.423
M0	77	40 (49%)	37 (51%)		
M1	30	13 (43%)	17 (57%)		
**Relapse**				.853	.356
Yes	94	45 (48%)	49 (52%)		
No	13	8 (62%)	5 (38%)		
**Metastasis**				.976	.323
Yes	11	7 (64%)	4 (36%)		
No	96	46 (48%)	50 (52%)		
**Ki67**				5.490	**.019** ^*^
≤.4	85	47 (55%)	38 (45%)		
>.4	22	6 (27%)	16 (73%)		
**EGFR**				4.042	**.044** ^*^
≤.75	73	41 (56%)	32 (44%)		
>.75	34	12 (35%)	22 (65%)		
**PD‐L1**				1.183	.277
≤.01	61	33 (54%)	28 (46%)		
>.01	46	20 (43%)	26 (57%)		

* *P* < 0.05 was considered significant.

In conclusion, our study demonstrated that MTHFR was upregulated in OV and the knock‐down of MTHFR inhibited the growth of OV cells both in vitro and in vivo. Moreover, MTHFR could suppress the ferroptosis through blocking K48‐linked ubiquitination of HMOX1 to stable HMOX1 (Figure 4K). Additionally, the high expression level of MTHFR was significantly associated with the poor prognosis of OV patients. In future work, it will be crucial to screen the potential role of MTHFR in cancer cells, which would pave the way for developing novel therapeutic drugs targeting MTHFR for diagnosis and therapeutic intervention of OV patients.

## CONFLICT OF INTEREST

The authors declare no conflict of interest.

## Supporting information

Supporting Information

Supporting Information

Supporting Information
