# Supplementary material for: MTHFR inhibits TRC8‐mediated HMOX1 ubiquitination and regulates ferroptosis in ovarian cancer
Source: Clin Transl Med. 2022 Sep 23;12(9):e1013. doi: 10.1002/ctm2.1013 (PMC9505752; doi:10.1002/ctm2.1013)
Supplement: Supplementary file 1 — Supporting Information [file CTM2-12-e1013-s003.docx]

**Supplementary Materials**

Material and methods

1.1 Cell culture and chemicals

Normal ovary cell line (IOSE80), OV cell lines (A2780, OVCAR3, SKOV3, TOV112D) and HEK293T cells were all obtained from Cancer Research Institute, Central South University. IOSE80 and SKOV3 cells were gown in RPMI 1640 media, while other cells were cultured in DMEM. All the media were supplemented with 10% fetal bovine serum and antibiotics containing 100 µg/mL streptomycin and 100 U/mL penicillin. Cells were cultured at 37 °C and 5% CO2. The chemicals used in this research including CDDP (APExBIO, A8321), Ferrostatin-1 (APExBIO, A4371), Z-VAD-FMK (APExBIO, A1902), Necrostatin-1 (APExBIO, A4213), MG132 (Selleckchem, S2619), Cycloheximide (Sigma, 01810).

1.2 Plasmids and transfection

MTHFR and HMOX1 plasmids were purchased from Sangon Biotech and cloned into the pLenti-CMV-GFP-Hygro vector. MTHFR shRNAs were purchased from Sangon Biotech and cloned into the pLKO.1-puro vector. TRC8 siRNAs were obtained from the company (Ribo Bio). MTHFR shRNA-1: CCGGCCGAAGTGAGTTTGGTGACTACTCGAGTAGTCACCAAACTCACTTCGGTTTTTG, AATTCAAAAACCGAAGTGAGTTTGGTGACTACTCGAGTAGTCACCAAACTCACTTCGG, MTHFR shRNA-2: CCGGAGTACGAGCTCCGGGTTAATTCTCGAGAATTAACCCGGAGCTCGTACTTTTTTG, AATTCAAAAAAGTACGAGCTCCGGGTTAATTCTCGAGAATTAACCCGGAGCTCGTACT. si-TRC8-1: GAACGAAGATGACAGTACA; si-TRC8-2: GTATCGAATTTACGGATTA. Lentivirus was packaged in HEK293T cells with the packaging plasmids pMD2.G and psPAX2. The media containing lentivirus was gathered after transfection for 48h. Then, the viruses were applied to infect the cells with polybrene (1 µg/ml). Two days later, the cells were grown with puromycin (1 µg/mL) for 4 days. Western blot was used to test the expression of MTHFR.

1.3 RNA extraction and reverse transcription polymerase chain reaction (RT-PCR)

Total RNA was extracted by TRIzol (Invitrogen). The Protein nucleic acid spectrophotometer (Beckman Coulter, United States) was applied to examine concentration of RNA. Then, the RNA was reverse transcribed to cDNA by PrimeScript™ RT kit. The RNA reaction was constructed by the SYBR Green kit and CFX96 touch real-time fluorescent quantitative PCR. 18S rRNA was applied as the internal reference. The 2−ΔΔCt method was used to test the relative expression levels of the target genes. The primer sequences applied in the study were showed in Supplemental Table 1.

1.4 Western Blot and Co-IP assay

Cells were washed with PBS and lysed in RIPA buffer containing protease inhibitor cocktail on ice. Protein concentrations were examined by the BCA protein kit (Thermo Scientific, 23225). Proteins were separated by SDS-PAGE, and electro-transferred to PVDF membranes. The PVDF membrane was incubated with a particular primary antibody at 4 °C overnight. After washing with TBST, the PVDF membrane was incubated with the appropriate secondary antibody for 1 h. Quantification of protein was detected with chemiluminescence reagent (Millipore, WBKLS0050). For Co-IP assays, the cell lysates were prepared in NETN buffer (20 mM Tris-HCl, pH 8.0, 100 mM NaCl, 1 mM EDTA, 0.5% Nonidet P-40) containing protease inhibitor. Then, the cell lysates were incubated with suitable antibodies and Protein A/G agarose beads for 2 h or overnight at 4 ℃. After washing the beads with NETN buffer for 4 times, the immuno-precipitate was resuspended in the 1×Loading Buffer. Western Blot assay were conducted to detect the expression of indicated proteins. For immunoprecipitation for ubiquitination, cells were lysed in the denaturing ubiquitination buffer (62.5 mM Tris-HCl (PH 6.8), 2% SDS, 10% glycerol, 20 mM NEM and 1 mM iodoacetamide) for 15 minutes on ice. Then, the lysates were subjected to immunoprecipitation with the indicated antibodies and blotted as indicated. The primary antibodies were used including: anti-MTFHR (Abcam, ab203786, 1:1000), anti-Actin (Santa Cruz Biotechnology, sc-8432, 1:2000), anti-HMOX1 (Proteintech, 10701-1-AP, 1:1000), anti-NRF2 (Proteintech, 66504-1-Ig, 1:1000), anti-Ubiquitin (Cell Signaling Technology, 3936, 1:1000), anti-K48-Polyubiquitin (Cell Signaling Technology, 12805, 1:1000), anti-K63-Polyubiquitin (Cell Signaling Technology, 12930, 1:1000), anti-TRC8 (Santa Cruz Biotechnology, sc-390347, 1:1000).

1.5 Colony formation assay

Cells were seeded in 6-well plates with a density of 800-1000 cells per well. For the next 14 days, cells were cultured at 37 °C in 5% CO2 and the media was renewed every 5 days. Then, cells were fixed with methanol. Colonies were stained with 0.3% crystal violet and counted. Results were normalized to plating efficiencies.

1.6 Cell viability assay

Cells (800-1000 per well) were seeded into 96- well plates. Cells growth was tested by CCK8 assay. After incubating with CCK8 for 2h, the cell number was examined by spectrofluorometer at wavelength OD450.

1.7 Reactive oxygen species (ROS) assay

The ROS levels wasere evaluated by a DCFDA / H2DCFDA (Abcam, ab113851) kit. Cells were seeded in the 6- well plates at an appropriate density. Incubated cells with the diluted DCFDA solution for 45 minutes at 37 ℃ in the dark. Then, the fluorescence intensity was examined using fluorescence microscope.

1.8 Iron assay

The ferrous iron (Fe2+) level was calculated by the iron assay kit (Abcam, ab83366). Cells were cultured in 96 well plates, and iron reducer and iron probe were added subsequently as previous reported[21]. After incubation, the absorbance was measured at 593 nm.

1.9 Malondialdehyde (MDA) assay

The content of MDA was performed by the Lipid Peroxidation MDA Assay Kit (Sigma, MAK085). 1 × 106 cells were homogenized with MDA Lysis Buffer containing BHT on ice. Collected the supernatant after 13000 × g for 10 minutes and added the TBA solution incubating at 95 ℃ for 60 minutes. The absorbance was measured at 532 nm.

1.10 Immunohistochemistry

The tissue microarrays of OV specimen were purchased from Outdo Biotech (Shanghai, China). The tissue sections were placed into EDTA solution after dewaxing and rehydration. Then, antigen retrieval was conducted at 100 °C for 30 min. A blocker was applied to cut off the activity of endogenous peroxidase for 15 min. Washing with PBS twice, the tissue sections were stained with MTHFR (Abcam, ab203786, 1:50) or HMOX1(Proteintech, 10701-1-AP, 1:200) at 4 °C overnight. Next day, washed twice with PBS and the tissue sections then incubated with a secondary antibody for 1 h at room temperature. The immunohistochemical signals were visualized with 3,3’-diaminobenzidine (DAB).

1.11 In vivo mice models

The female BALB/c nude mice (nu/nu, aged 4-6 weeks) were used. 5×106 A2780 cells were suspended in 50 μl matrigel, and then subcutaneously injected into the mouse (seven mice per group). Tumor size s wasere monitored every two days. Tumor volume was calculated by the following formula: tumor volume = (length × width2)/2. The study was approved by the Animal Ethics Committee of Central South University.

1.12 Statistical analyses

All data were presented with mean± standard deviation (SD), and analyzed by the student’s t test. All the experiments were repeated at least three times independently. The association between clinicopathological data and the MTHFR and HMOX1 scores was performed using chi-squared test. Survival curves were analyzed by the log-rank test. SPSS 20.0 and GraphPad Prism 8 were applied for statistical analysis. P < 0.05 was considered significant.
