# Supplementary figures and images for: MTHFR inhibits TRC8‐mediated HMOX1 ubiquitination and regulates ferroptosis in ovarian cancer
Source: Clin Transl Med. 2022 Sep 23;12(9):e1013. doi: 10.1002/ctm2.1013 (PMC9505752; doi:10.1002/ctm2.1013)

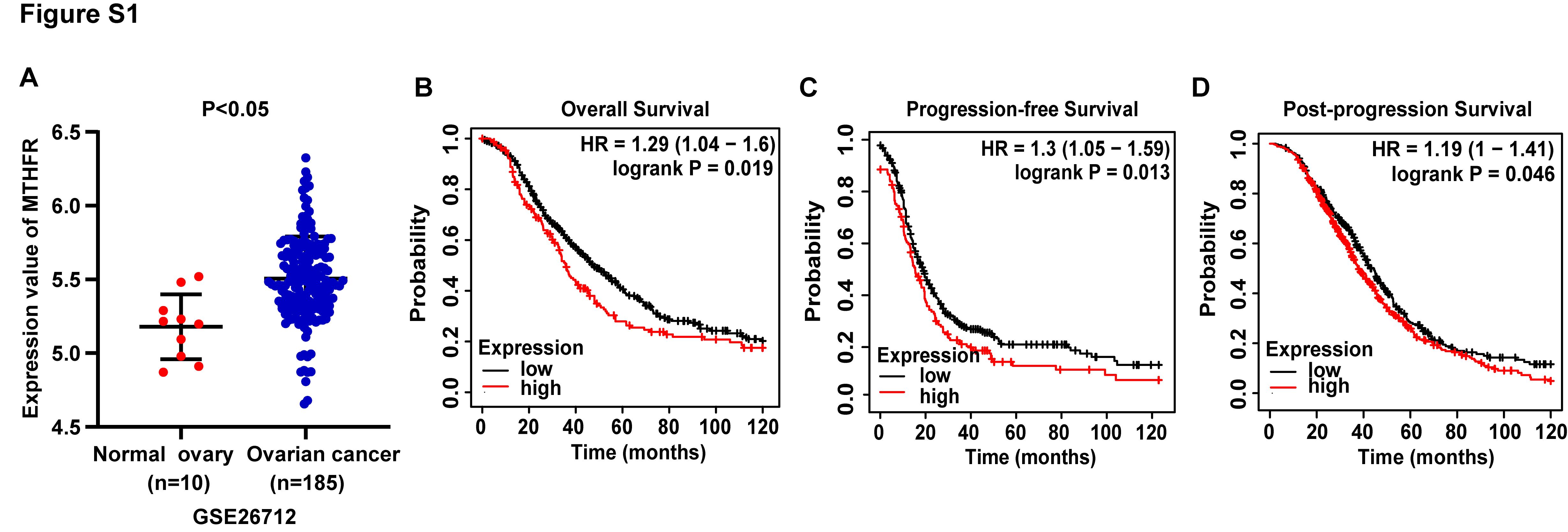

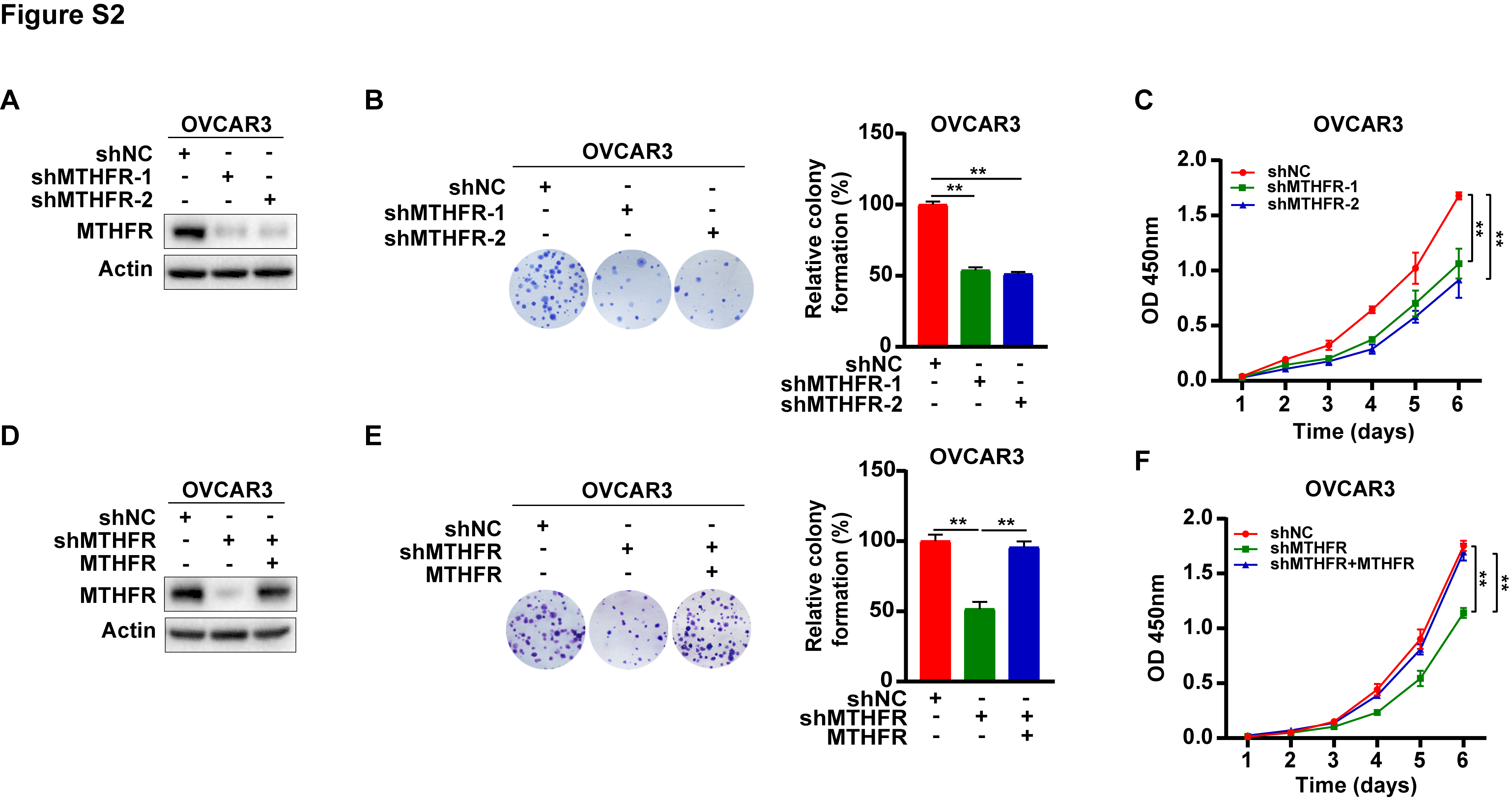

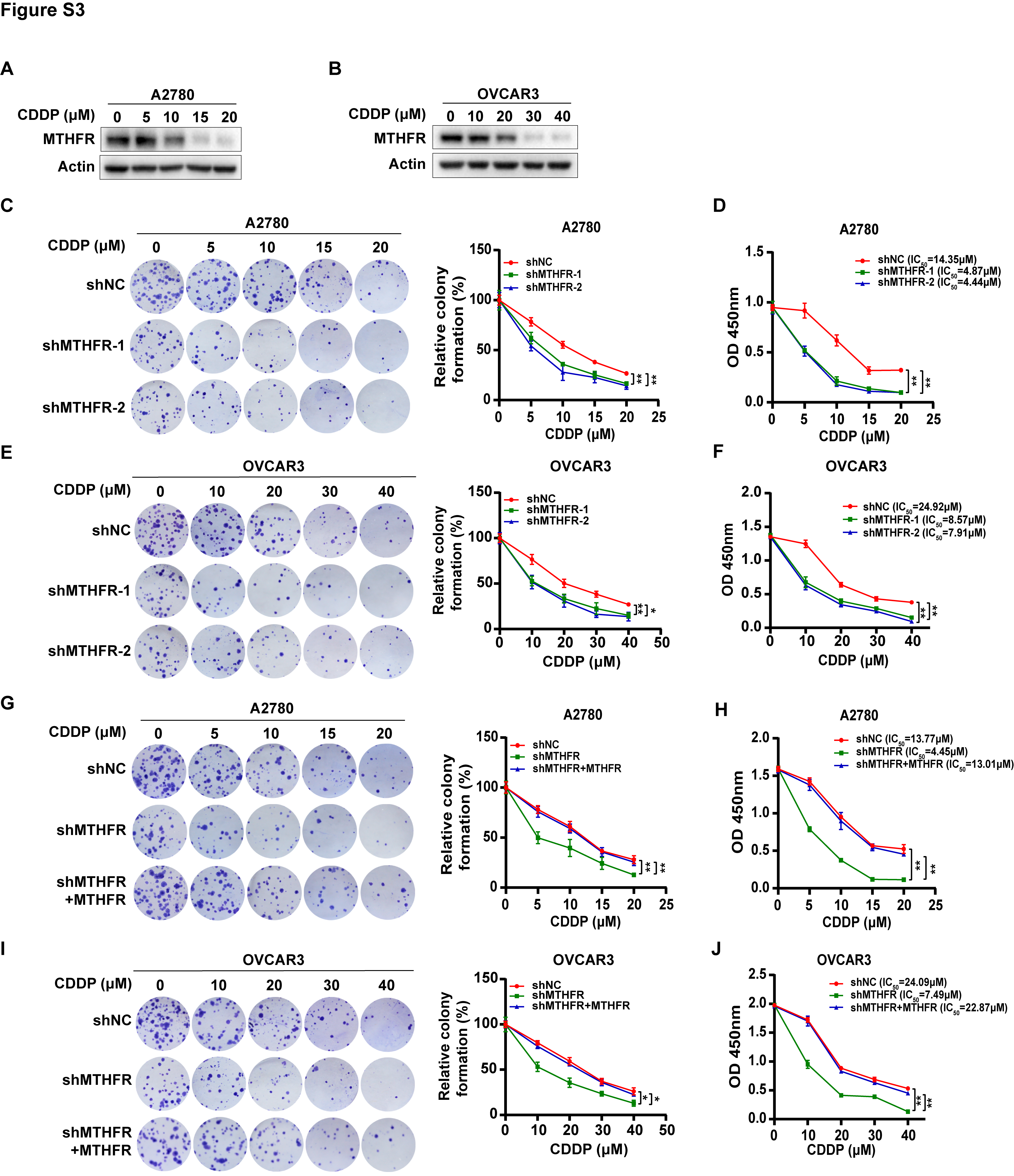

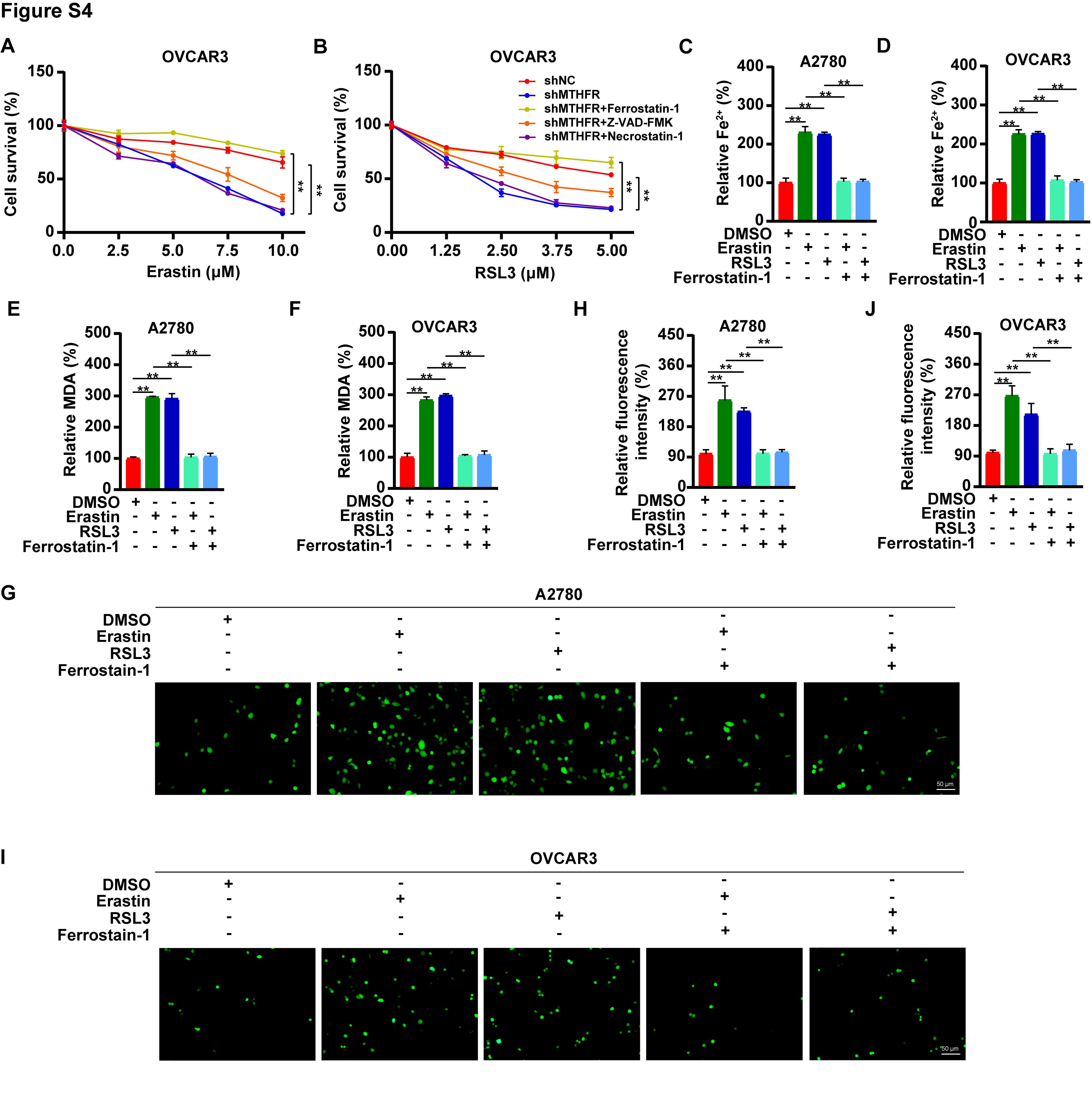

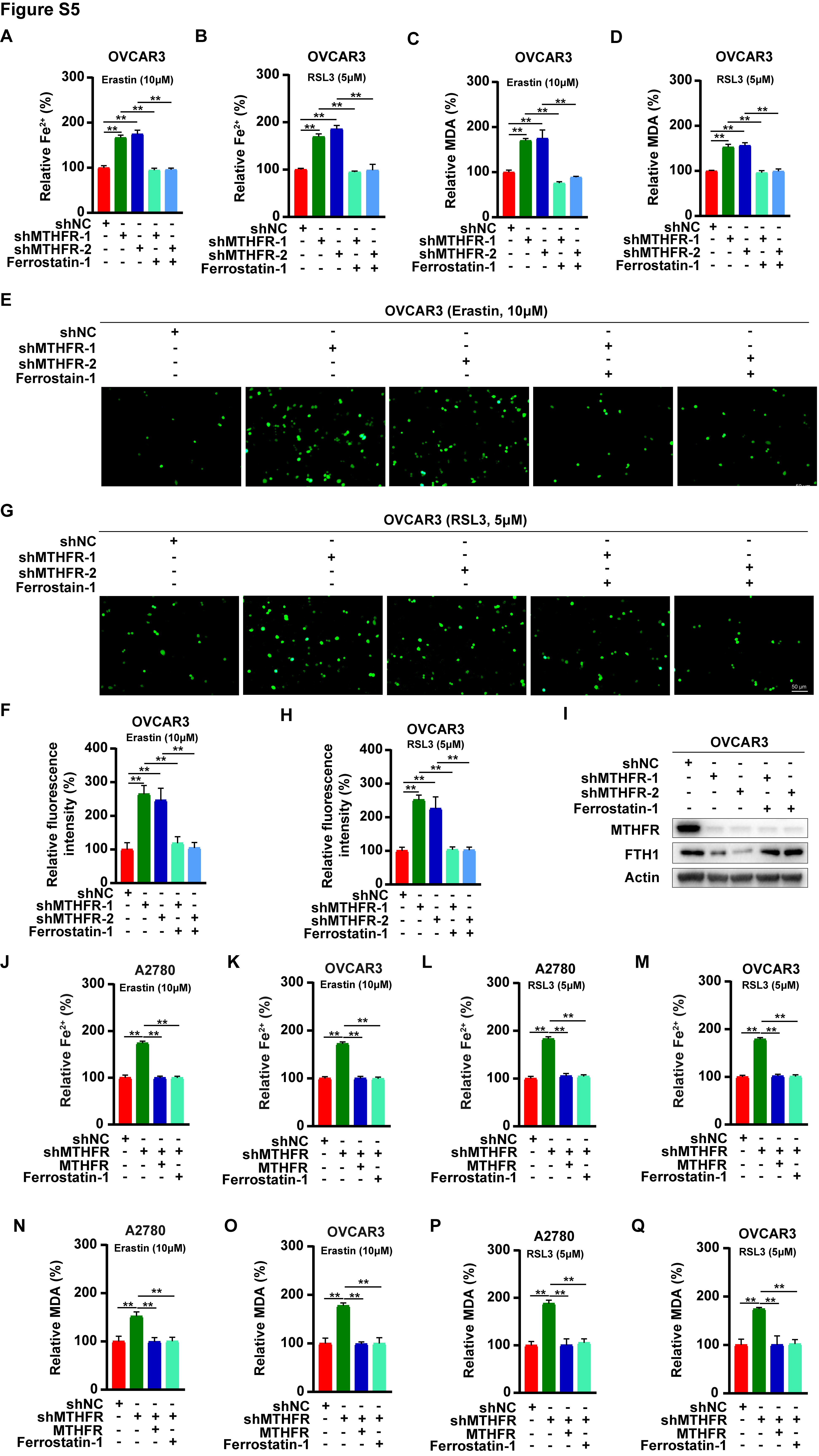

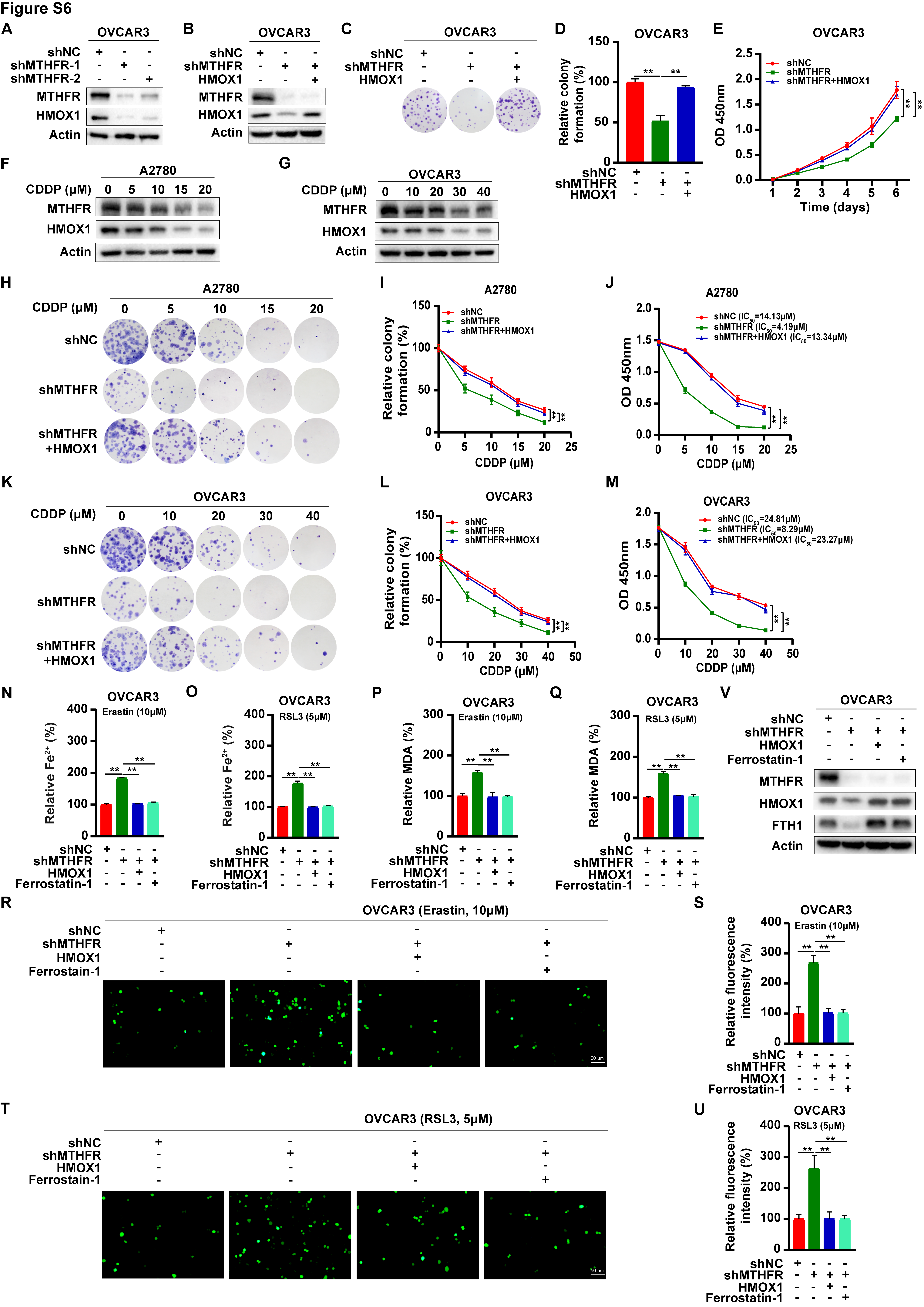

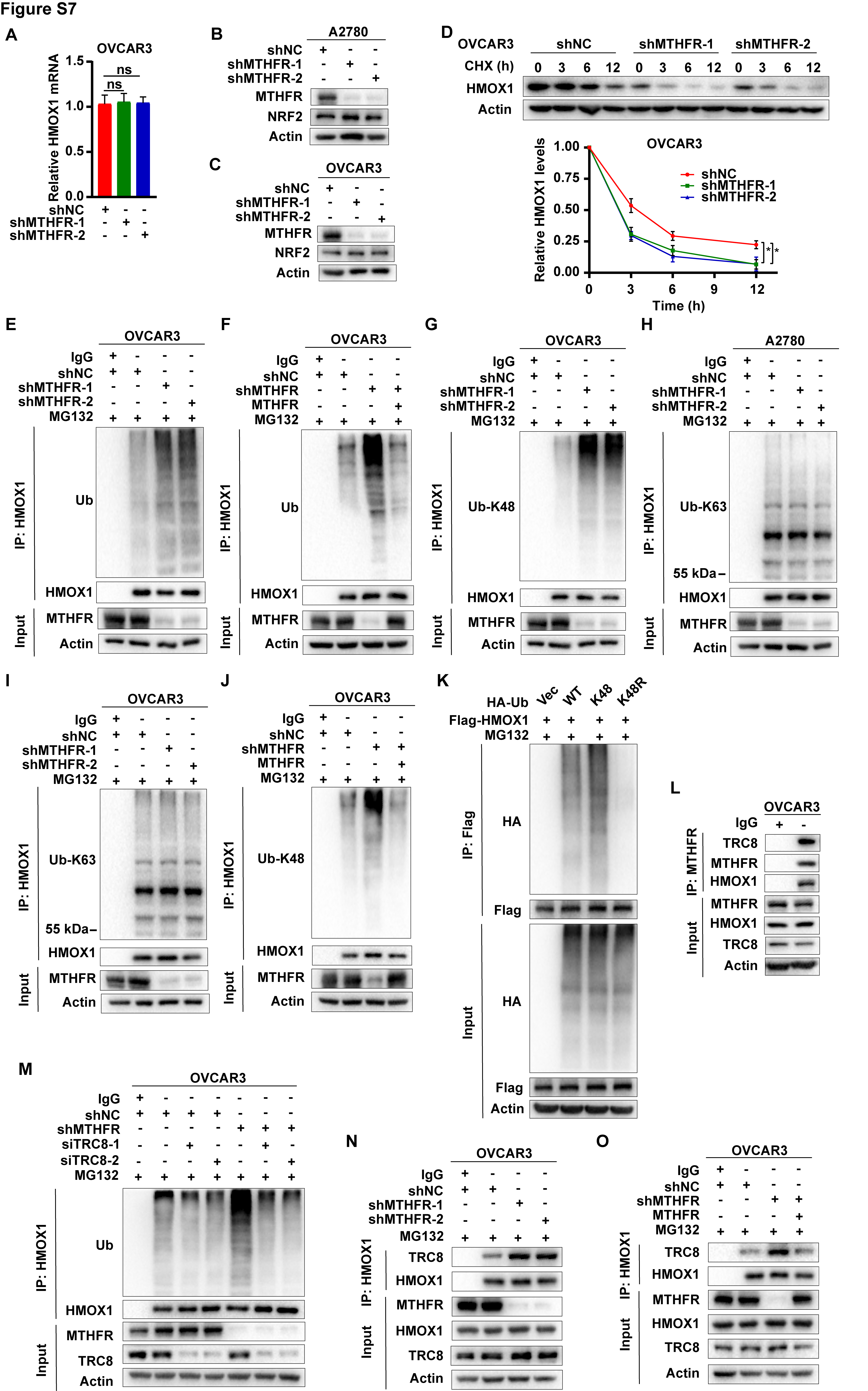

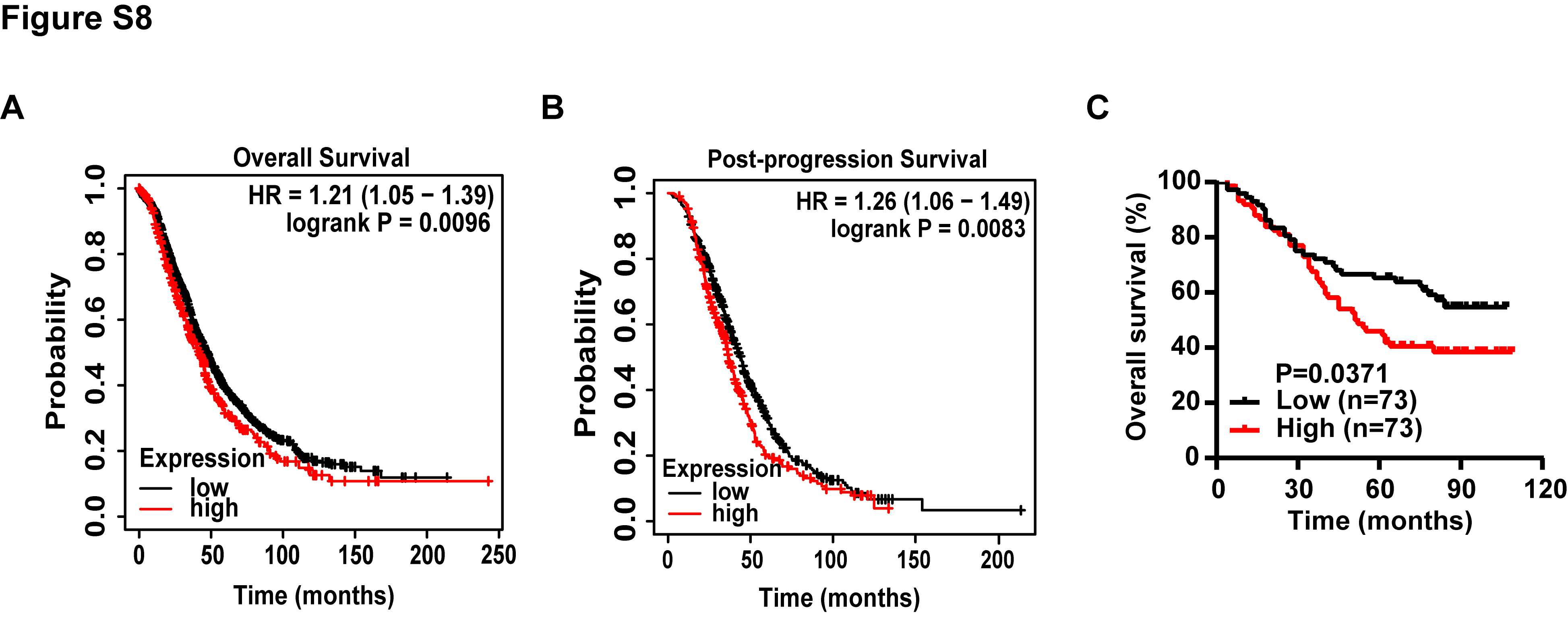

Supplement: Supplementary file 2 — Supporting Information [file CTM2-12-e1013-s002.docx]
