# Supplementary material for: MTHFR inhibits TRC8‐mediated HMOX1 ubiquitination and regulates ferroptosis in ovarian cancer
Source: Clin Transl Med. 2022 Sep 23;12(9):e1013. doi: 10.1002/ctm2.1013 (PMC9505752; doi:10.1002/ctm2.1013)
Supplement: Supplementary file 3 — Supporting Information [file CTM2-12-e1013-s001.docx]

Table S1. The differentially expressed proteins in MTHFR-interfered A2780 cells.

| Protein | Foldchange | Protein | Foldchange | Protein | Foldchange | Protein | Foldchange |
| --- | --- | --- | --- | --- | --- | --- | --- |
| TITIN | 55.4500453 | NUBP2 | 6.70339872 | MPP10 | 4.95240019 | WDR5 | 4.15587953 |
| RM13 | 50.5638708 | STAT6 | 6.68964701 | THTM | 4.94773689 | INT14 | 4.14092064 |
| EFNMT | 46.6101174 | HIF1N | 6.63616599 | SCRN2 | 4.91590164 | IF6 | 4.13264632 |
| RPP40 | 30.104482 | EPN1 | 6.55904116 | ADDA | 4.90005342 | INT7 | 4.13215083 |
| ACO13 | 25.2433951 | MAN1 | 6.55864703 | UBE4B | 4.89298059 | TBG1 | 4.09992002 |
| NHRF3 | 20.1630628 | MK14 | 6.5461544 | SPF27 | 4.88435267 | SC61G | 4.09397129 |
| ACSF2 | 17.2849828 | C2D1A | 6.48294705 | PLRG1 | 4.85303253 | LYSM2 | 4.07166835 |
| AP4E1 | 15.0373113 | S10A4 | 6.36789703 | LRSM1 | 4.8104934 | LTOR1 | 4.07110418 |
| THIK | 12.5316334 | HEXA | 6.28091902 | PLD3A | 4.75971635 | THOP1 | 4.06591735 |
| CHMP7 | 11.446653 | SAFB2 | 6.26772168 | FRIL | 4.72055557 | RBGPR | 4.06362682 |
| UBL5 | 11.3323391 | AQR | 6.2406193 | CISD1 | 4.71665508 | PPP6 | 4.04842277 |
| KS6A1 | 11.1906916 | MGME1 | 6.21220716 | CETN2 | 4.58389037 | MSRB3 | 4.02629786 |
| FXR2 | 11.1075387 | TBA1C | 6.17678776 | FHOD1 | 4.5517912 | RRAS | 4.02028374 |
| PAK4 | 11.0434328 | AKA12 | 6.07540887 | CPSF2 | 4.55134029 | NUBP1 | 3.99921837 |
| ZNT7 | 10.8273161 | CBX8 | 6.07331238 | ATD3B | 4.5446545 | DAG1 | 3.99196352 |
| HPS6 | 10.7909753 | PBIP1 | 6.05158882 | UBE4A | 4.53349195 | RTCA | 3.97802684 |
| 44812 | 10.6562651 | QCR9 | 5.99709682 | PIPNA | 4.52444123 | PPIF | 3.94151367 |
| RT09 | 10.6400351 | UT14A | 5.99510064 | EH1L1 | 4.50037835 | NOP53 | 3.93507931 |
| CCD25 | 10.5606362 | TM9S2 | 5.90253956 | CHCH1 | 4.49703877 | TFB1M | 3.92012742 |
| SAFB1 | 9.78138264 | RRP1B | 5.89288106 | AS3MT | 4.49292485 | CSN7A | 3.91907624 |
| GGH | 9.24951134 | SYMPK | 5.88919197 | ARAP1 | 4.49253051 | COXM2 | 3.91445422 |
| PATL1 | 9.22892808 | BAX | 5.81587652 | RTKN | 4.47991953 | GAMT | 3.90985122 |
| HMGN5 | 8.94983591 | GOG8S | 5.6793728 | CAB39 | 4.46442718 | AP2S1 | 3.89954338 |
| CPSF1 | 8.69509443 | SEPT5 | 5.54369379 | GLCNE | 4.4637583 | CAP2 | 3.88637831 |
| TM214 | 8.38950362 | OSBP1 | 5.43100396 | CSPG4 | 4.46032335 | CAVN1 | 3.85595069 |
| TMUB1 | 8.05694996 | VAMP3 | 5.39147613 | CLIP1 | 4.45900549 | PRIO | 3.85220803 |
| ISOC1 | 8.03719271 | PLPHP | 5.37379068 | ITB6 | 4.44745203 | OXA1L | 3.8407291 |
| H10 | 7.87455539 | GLMN | 5.3558234 | THUM3 | 4.35197584 | HNRLL | 3.8206833 |
| EXOS8 | 7.65867829 | RPB3 | 5.18059148 | UBP24 | 4.32743093 | FL2D | 3.8191148 |
| RING1 | 7.53858849 | GDE | 5.17673361 | PSF2 | 4.32196475 | PUM3 | 3.79916025 |
| PHF6 | 7.31985062 | STXB2 | 5.16816817 | ACTG | 4.3219126 | PEX14 | 3.77857223 |
| NOP10 | 7.29952334 | KPRA | 5.15152592 | MVP | 4.29927415 | S10A2 | 3.76661354 |
| MOD5 | 7.28721315 | STX12 | 5.13607082 | RDH10 | 4.28772304 | MYH14 | 3.76271031 |
| GBP2 | 7.13126483 | RM03 | 5.13219565 | SEM3C | 4.27896291 | RT35 | 3.75343585 |
| NIBA2 | 7.11969146 | FRG1 | 5.10480764 | CPT2 | 4.26387892 | CERS2 | 3.73964166 |
| CCG6 | 7.11040651 | NHLC2 | 5.06511309 | DPOE3 | 4.23895138 | KPRB | 3.73442665 |
| DDX24 | 7.02201206 | PTH2 | 5.01403845 | FLOT1 | 4.23619168 | DPOD1 | 3.72412615 |
| MTA70 | 6.78601289 | ODPB | 4.99762065 | PHAR4 | 4.22193313 | DYR | 3.71951322 |
| PRRC1 | 6.75969266 | OSGEP | 4.96222304 | SPRY4 | 4.15608362 | CPNE1 | 3.70529229 |

| Protein | Foldchange | Protein | Foldchange | Protein | Foldchange | Protein | Foldchange |
| --- | --- | --- | --- | --- | --- | --- | --- |
| THEM6 | 3.69077631 | PDXK | 3.43495043 | C1TM | 3.17312709 | SCRB2 | 3.00574879 |
| IDH3G | 3.68697855 | SPB9 | 3.42358049 | SCAM1 | 3.16570715 | NFU1 | 2.99635059 |
| IRF3 | 3.67350053 | STK39 | 3.41947977 | RAP1A | 3.16411255 | SEH1 | 2.9931853 |
| ECI1 | 3.65996587 | HSDL2 | 3.39319704 | NAGAB | 3.15479651 | STXB1 | 2.98864776 |
| SEN34 | 3.65525478 | E2AK4 | 3.39240694 | CD276 | 3.14827088 | AL1B1 | 2.98595944 |
| NDUA9 | 3.64732607 | DNS2A | 3.39017562 | GALE | 3.14093735 | PLD3 | 2.98319381 |
| ARK72 | 3.64227976 | UBP48 | 3.37628727 | PML | 3.13974635 | AT1A1 | 2.98075628 |
| PLBL2 | 3.63004516 | HNRH2 | 3.36982769 | AL1A1 | 3.12679931 | CDC42 | 2.97343945 |
| SCYL1 | 3.6280438 | TRI25 | 3.35816974 | MVD1 | 3.12442828 | RAB4A | 2.97320324 |
| ERP44 | 3.62728087 | HCDH | 3.33513039 | KV112 | 3.1168793 | MEP50 | 2.96020847 |
| ATF1 | 3.62043013 | CDC27 | 3.32421837 | CBWD1 | 3.11645464 | AAAT | 2.95895354 |
| MIRO2 | 3.61234912 | APC7 | 3.32048554 | GALK1 | 3.11551571 | GCDH | 2.9583868 |
| ACTC | 3.60767012 | CN37 | 3.31361407 | PTN1 | 3.10472418 | WDR61 | 2.95478106 |
| SMRC2 | 3.59912603 | AT2B4 | 3.30949573 | BCAT2 | 3.10190088 | MMS19 | 2.95380021 |
| RAB10 | 3.59583459 | LTOR5 | 3.30398027 | ECH1 | 3.09978709 | JUPI1 | 2.94775043 |
| PPIL4 | 3.59482579 | ABR | 3.29505343 | VAT1 | 3.09776925 | SYIM | 2.93907646 |
| OAT | 3.58624798 | PURA2 | 3.29422222 | TBB6 | 3.08689851 | PRUN1 | 2.9382877 |
| TBK1 | 3.57570027 | DBLOH | 3.28183348 | MOB1A | 3.08496177 | H2AW | 2.93674699 |
| SCFD1 | 3.57520325 | ADT1 | 3.27293772 | CCD86 | 3.08216065 | RAB32 | 2.9362183 |
| SSU72 | 3.54363647 | BRE1A | 3.27246214 | SNTB2 | 3.08057372 | CRTAP | 2.93192168 |
| GOLI4 | 3.53259835 | SCO1 | 3.27126893 | RAB2A | 3.0792362 | NADC | 2.92291249 |
| I2BP2 | 3.52704595 | EHD4 | 3.26408369 | RPAC1 | 3.07431255 | CLAP2 | 2.92195314 |
| ARMT1 | 3.52585466 | PMVK | 3.26294015 | AAPK1 | 3.06966216 | GBF1 | 2.92164361 |
| S12A2 | 3.51948439 | GCP60 | 3.25519392 | DDX41 | 3.06052448 | RM46 | 2.92007105 |
| NT5D1 | 3.51875692 | RN115 | 3.25175956 | PXL2A | 3.05977494 | GON7 | 2.91946034 |
| ACTY | 3.51719295 | ORC3 | 3.24876802 | NDUBB | 3.05560578 | MARH5 | 2.91644237 |
| LRBA | 3.50098414 | DCMC | 3.24234244 | F1142 | 3.05527544 | MA2B1 | 2.90862069 |
| KBL | 3.49813 | IDH3B | 3.23767638 | GNPI1 | 3.05463044 | DOCK7 | 2.90124035 |
| CK068 | 3.4925343 | NIPS2 | 3.23010638 | SVIL | 3.04917972 | ETFD | 2.90114932 |
| THIL | 3.49016121 | HAUS6 | 3.22540453 | HXK1 | 3.04560795 | DPOD3 | 2.89615285 |
| IF4E | 3.48219109 | BMS1 | 3.22436339 | GBRAP | 3.03744662 | MPZL1 | 2.89610544 |
| IPO9 | 3.47877558 | KDM1A | 3.22265483 | UGPA | 3.03580685 | ADT2 | 2.8951679 |
| PP4P1 | 3.47476168 | YTDC1 | 3.21412964 | RPE | 3.0314357 | ZPR1 | 2.89420021 |
| CND3 | 3.4703417 | RM02 | 3.19767442 | TYSY | 3.02660407 | GYS1 | 2.89056107 |
| CSN7B | 3.46750435 | CI016 | 3.19698251 | UBE2A | 3.02588893 | BPNT1 | 2.88930667 |
| RM41 | 3.46072516 | RPP30 | 3.19318212 | S10A1 | 3.01901117 | FA98A | 2.87654926 |
| PSA2 | 3.45394737 | DCUP | 3.18858384 | H15 | 3.01740298 | ACDSB | 2.87594761 |
| GMPPB | 3.4532 | NDUS2 | 3.18794159 | ATPF1 | 3.01626723 | NFKB2 | 2.87339507 |
| SUMO2 | 3.43921964 | RNZ2 | 3.17622236 | ERAP1 | 3.0118624 | ECHB | 2.87237156 |

| Protein | Foldchange | Protein | Foldchange | Protein | Foldchange | Protein | Foldchange |
| --- | --- | --- | --- | --- | --- | --- | --- |
| NEK9 | 2.86908078 | SMD1 | 2.76967463 | RT30 | 2.64827089 | MYOF | 2.55907629 |
| KITH | 2.86493861 | COMD3 | 2.76947938 | CMC2 | 2.64698511 | CIRBP | 2.5539411 |
| MMTA2 | 2.86245601 | OCAD1 | 2.7670297 | CPPED | 2.64632841 | HNRH3 | 2.55282367 |
| EI2BE | 2.85886709 | MAVS | 2.75897326 | RDH13 | 2.64355112 | APT | 2.54947614 |
| SRS11 | 2.8556891 | ATP5L | 2.75853801 | LIN7C | 2.64262825 | SLTM | 2.54856324 |
| ATG3 | 2.85379587 | MTNA | 2.75484135 | MP2K4 | 2.64146053 | CATC | 2.54800039 |
| EF1A2 | 2.85379425 | RL18 | 2.75064134 | KAD4 | 2.64129357 | SDHA | 2.54720538 |
| TMED7 | 2.85296811 | DPYL2 | 2.74299962 | FAM3C | 2.63741403 | GSLG1 | 2.54702693 |
| PPIL1 | 2.84697529 | AP2A1 | 2.74252148 | SELH | 2.63739337 | RALY | 2.54385324 |
| MAGT1 | 2.84085288 | SAC1 | 2.74033687 | GHC1 | 2.63590131 | HM13 | 2.54248507 |
| NELFB | 2.8388481 | QKI | 2.73638095 | PDCD6 | 2.63579278 | RM09 | 2.54216662 |
| PYGL | 2.83774353 | ITA5 | 2.73616229 | SYFA | 2.63351665 | ARP5L | 2.53912908 |
| UBF1 | 2.82702081 | SMCE1 | 2.72301703 | ECHA | 2.63198129 | DKC1 | 2.53832495 |
| ISCU | 2.82544379 | TPD52 | 2.72037456 | LIMS4 | 2.62834557 | ATOX1 | 2.53780705 |
| NALP2 | 2.82079156 | GNS | 2.71470262 | PSA | 2.62055587 | RFC2 | 2.53578766 |
| FIP1 | 2.82076402 | SYLC | 2.71435071 | PSB7 | 2.620014 | PDXD1 | 2.53427128 |
| ASAH1 | 2.81933564 | TMM43 | 2.71287187 | COPG2 | 2.61804175 | GNAI2 | 2.53422511 |
| 5NTC | 2.81554728 | NNTM | 2.71220572 | PIN1 | 2.61550889 | PCP | 2.53395238 |
| DUS3 | 2.81507033 | RBM42 | 2.70863656 | CHM2A | 2.61518565 | SNX1 | 2.53381957 |
| MYDGF | 2.80829619 | NCS1 | 2.70860614 | P3C2A | 2.61285999 | SMC1A | 2.53214893 |
| ACADV | 2.80691807 | ZW10 | 2.70722685 | RM16 | 2.61099478 | FAAA | 2.53101093 |
| NU153 | 2.80623267 | AMPD2 | 2.7030856 | TMED5 | 2.60991924 | GLYG | 2.52955583 |
| P4HA2 | 2.80420485 | HEAT1 | 2.69214732 | LONM | 2.60796521 | CAZA2 | 2.52365434 |
| RM22 | 2.80185553 | MPRI | 2.68004137 | IF4A2 | 2.60344356 | ELAV1 | 2.52079119 |
| CATD | 2.80087848 | RL36 | 2.67770441 | RTRAF | 2.60251255 | FRIH | 2.52058003 |
| TP53B | 2.80025052 | KS6A3 | 2.67599989 | EHD1 | 2.5976708 | RPB1 | 2.52043092 |
| SKAP | 2.79975219 | SURF4 | 2.67577449 | IDHP | 2.59266818 | NDUA4 | 2.51755252 |
| EI2BD | 2.79402177 | PRP4B | 2.67136131 | AP1M1 | 2.59115877 | INO1 | 2.51578491 |
| FLII | 2.7939828 | PDIP3 | 2.67023847 | HNRPF | 2.58981314 | SMHD1 | 2.51345684 |
| TAF5 | 2.79230931 | IF2B3 | 2.66787672 | NUDT3 | 2.58911089 | SPY4 | 2.50958383 |
| ARLY | 2.78857837 | SEC63 | 2.66739249 | LANC2 | 2.58820912 | PLGT3 | 2.50429424 |
| RBBP9 | 2.78637183 | TTC9C | 2.66530512 | TPP2 | 2.58608764 | PFKAP | 2.50339949 |
| IMA7 | 2.78582067 | IPO4 | 2.66259015 | BIN1 | 2.58531432 | VATH | 2.50320789 |
| ACSL1 | 2.78479604 | LTOR3 | 2.66197351 | CATB | 2.58446733 | MP2K2 | 2.50094084 |
| BACH | 2.78235131 | 2A5A | 2.66150038 | FYCO1 | 2.57129882 | NSDHL | 2.50068193 |
| HARS1 | 2.77411118 | SAM50 | 2.65829883 | SH3G1 | 2.56772929 | PSMG1 | 2.49193776 |
| SDF2L | 2.77377139 | RL7A | 2.65258443 | AP3B1 | 2.56505978 | SH24A | 2.49103385 |
| NR2E3 | 2.77204256 | PHB | 2.65234765 | SYCC | 2.56332005 | CCD22 | 2.49090672 |
| PFKAL | 2.77131535 | DNPH1 | 2.65213581 | LYPA2 | 2.56289275 | MBNL1 | 2.48844522 |

| Protein | Foldchange | Protein | Foldchange | Protein | Foldchange | Protein | Foldchange |
| --- | --- | --- | --- | --- | --- | --- | --- |
| QOR | 2.48839911 | ATP5H | 2.41500115 | AGM1 | 2.35070372 | FERM2 | 2.29763733 |
| ANM3 | 2.4881983 | RRBP1 | 2.41498304 | CNOT1 | 2.35047177 | WASC4 | 2.2975339 |
| GRWD1 | 2.47791023 | UAP1 | 2.4145449 | PSMD5 | 2.34765388 | FBX2 | 2.29741898 |
| TRA2B | 2.47530864 | P5CS | 2.41367998 | TPPC3 | 2.34604206 | ASNS | 2.29726494 |
| GFPT2 | 2.47257098 | NEMO | 2.41221316 | RFA2 | 2.34576867 | ADRO | 2.29523835 |
| PSB3 | 2.46967516 | CIP2A | 2.41157973 | KRR1 | 2.342835 | TIM44 | 2.29511203 |
| UCHL1 | 2.46624949 | VKOR1 | 2.40858639 | STXB3 | 2.34116798 | PROF3 | 2.2930026 |
| ILKAP | 2.46456231 | VPS16 | 2.40787211 | ARPC4 | 2.34081167 | NCKP1 | 2.29209118 |
| ESYT2 | 2.4634587 | TWF2 | 2.40338621 | MPPB | 2.34014979 | PRPS2 | 2.29121575 |
| UB2G2 | 2.46249652 | H2A3 | 2.39982087 | DSG2 | 2.33976981 | PP1B | 2.29042782 |
| ACTN4 | 2.4615152 | NXF1 | 2.39386973 | PPIC | 2.33787313 | SPRE | 2.29029301 |
| HYOU1 | 2.45813091 | TDRKH | 2.38548296 | TCOF | 2.33482252 | TMA16 | 2.28814997 |
| MCA3 | 2.45682487 | GSH0 | 2.38384956 | SUCA | 2.33288062 | STX17 | 2.28803512 |
| BAG3 | 2.45530018 | CLPB | 2.38275028 | ATLA3 | 2.33242103 | ELP3 | 2.28499931 |
| RAB1A | 2.45484281 | SCOT1 | 2.38234366 | WASH6 | 2.33160337 | NPC2 | 2.2836122 |
| RAB21 | 2.45475246 | SARNP | 2.37775989 | DOHH | 2.32943203 | TBB4A | 2.28344583 |
| LSM5 | 2.45366333 | CNDP2 | 2.37689518 | PSPC1 | 2.32842288 | RL8 | 2.28119653 |
| RM21 | 2.4519108 | PP4C | 2.37635441 | FBRL | 2.32808455 | KIF4A | 2.28065655 |
| OSTC | 2.45118873 | QCR1 | 2.3760311 | VP13D | 2.32604528 | SMD3 | 2.27793383 |
| UN45A | 2.450497 | MFAP1 | 2.37565076 | ZC11A | 2.3224593 | NDK3 | 2.27783691 |
| TMM11 | 2.44937512 | RAB13 | 2.37335853 | MAIP1 | 2.3221971 | COPE | 2.27673565 |
| FKBP8 | 2.44854881 | LEGL | 2.3725406 | DNM1L | 2.31905657 | MYO1C | 2.2763232 |
| DHX16 | 2.44673017 | THOC2 | 2.37134303 | SAE2 | 2.31527825 | ELOC | 2.27491921 |
| ADT3 | 2.44570406 | TECR | 2.36902615 | SPB1 | 2.31516941 | EDC4 | 2.27434423 |
| COASY | 2.44518121 | MPU1 | 2.36814451 | PGM2 | 2.3125257 | SAHH | 2.27274566 |
| NUDT5 | 2.44445105 | PSME1 | 2.36729732 | GRTP1 | 2.312259 | ILF2 | 2.27067791 |
| PHB2 | 2.43916385 | NTPCR | 2.36632039 | CLN6 | 2.31174686 | PIPNB | 2.27041925 |
| SLIRP | 2.43850378 | MLEC | 2.36109862 | HMGA1 | 2.30961039 | PP4R1 | 2.2695162 |
| RL38 | 2.43745152 | DNPEP | 2.36028717 | CSKI2 | 2.30933277 | SEC20 | 2.26899955 |
| TMM70 | 2.43717774 | SRRM2 | 2.35937246 | MCTS1 | 2.3048048 | LYPA1 | 2.26859062 |
| DAAF5 | 2.43485977 | PGTB1 | 2.35904684 | STK26 | 2.30357928 | EPN4 | 2.26817885 |
| AIFM1 | 2.43243375 | UGGG1 | 2.35761727 | LETM1 | 2.30310302 | ABCF3 | 2.26800467 |
| S2535 | 2.43073295 | AP2A2 | 2.35744066 | TF3C5 | 2.30198874 | TOM40 | 2.26694676 |
| PACN2 | 2.42703689 | OPA1 | 2.3573907 | EMC2 | 2.30153172 | AAAS | 2.26601095 |
| C19L1 | 2.4253973 | MCM5 | 2.35720698 | RT26 | 2.30096189 | PDS5B | 2.26597829 |
| CSK22 | 2.42099567 | CISD2 | 2.35595314 | SDHB | 2.30077506 | TM1L2 | 2.26456145 |
| CTBP1 | 2.41962888 | DNJC1 | 2.35367746 | TXTP | 2.30054964 | AL3A2 | 2.262764 |
| DLDH | 2.41668749 | COX2 | 2.3519921 | NDRG3 | 2.29870824 | ATPK | 2.26254876 |
| VAMP7 | 2.41580198 | ZN790 | 2.35142677 | COPZ1 | 2.29845828 | NUMA1 | 2.26226636 |

| Protein | Foldchange | Protein | Foldchange | Protein | Foldchange | Protein | Foldchange |
| --- | --- | --- | --- | --- | --- | --- | --- |
| NDUS7 | 2.2617127 | RT23 | 2.20664769 | PRS6A | 2.16591269 | ICAL | 2.12396208 |
| HCD2 | 2.26084461 | AMPB | 2.20620469 | GLSK | 2.16459268 | NOP56 | 2.12286059 |
| SC23B | 2.25726276 | ECHM | 2.20593592 | PEPD | 2.16447872 | PSMD7 | 2.12280702 |
| CDK1 | 2.25684242 | MBOA7 | 2.20473979 | BROX | 2.16411312 | TR112 | 2.12255032 |
| CBPD | 2.25631673 | NDUV1 | 2.20430009 | DDAH2 | 2.16386837 | VATB2 | 2.12026562 |
| STRN4 | 2.25567445 | KINH | 2.2041997 | UCHL3 | 2.16383289 | CCAR1 | 2.11936779 |
| SNX2 | 2.25531648 | 44810 | 2.2039941 | CBX1 | 2.16318622 | HMGB2 | 2.11903669 |
| UHRF2 | 2.25383053 | HMCS1 | 2.20112074 | DCA13 | 2.16260292 | SSDH | 2.11781046 |
| MCMBP | 2.25158507 | UBCP1 | 2.19896559 | AP3S1 | 2.16209703 | MAT2B | 2.11758236 |
| PCYOX | 2.24402819 | GLYM | 2.19725931 | TPM2 | 2.16061238 | RCC1 | 2.1173821 |
| TIGAR | 2.24275787 | NUP35 | 2.19652469 | SYDC | 2.15995099 | MANF | 2.11528089 |
| MRE11 | 2.24234471 | RN181 | 2.19436408 | PSB2 | 2.15792309 | SRSF2 | 2.11397657 |
| FKB10 | 2.23764065 | OST48 | 2.19275102 | UBQL1 | 2.15434411 | PRDX5 | 2.11347418 |
| PBDC1 | 2.23679683 | IPP2 | 2.19121447 | NU205 | 2.15083075 | TBC15 | 2.11207238 |
| DNMBP | 2.23569139 | SC22B | 2.18911433 | UBP10 | 2.15025823 | ACPM | 2.11196408 |
| SERA | 2.23289316 | BAG6 | 2.1867753 | SGMR1 | 2.15006589 | RS30 | 2.11193195 |
| ODPA | 2.23285335 | MYL1 | 2.18636305 | UBE2S | 2.1492778 | NAT10 | 2.11171324 |
| DNJB1 | 2.2325843 | MIC13 | 2.18628754 | KIF23 | 2.14922373 | PRS6B | 2.11169566 |
| RL28 | 2.23132492 | CUL1 | 2.18479186 | AGRE5 | 2.14885608 | ECI2 | 2.11118545 |
| RBM3 | 2.23122249 | VDAC1 | 2.18421053 | TLN1 | 2.14646316 | MYG1 | 2.1108232 |
| CSN6 | 2.2280142 | LA | 2.18328754 | XPOT | 2.14615524 | UBE2H | 2.10955264 |
| ATPD | 2.22653722 | BLMH | 2.18295326 | RENT1 | 2.14566432 | DDX46 | 2.10948268 |
| GNAS2 | 2.22533886 | PSD11 | 2.18294294 | GOGA3 | 2.1447717 | TALDO | 2.1089026 |
| ORC2 | 2.22328524 | PYRG2 | 2.18225463 | NDUB3 | 2.14458201 | ICLN | 2.10853432 |
| CPNS1 | 2.22319532 | ACOX1 | 2.182178 | RAB1B | 2.14384255 | ZCCHL | 2.10833524 |
| BLVRB | 2.22304227 | RFA3 | 2.18115712 | UFC1 | 2.14266255 | CYB5B | 2.1082683 |
| TBCD | 2.2228931 | SSBP | 2.18085106 | NUCB2 | 2.14168432 | SYYM | 2.10723501 |
| C56D2 | 2.22285048 | CTR9 | 2.18001281 | AUP1 | 2.14049795 | UBAP2 | 2.10713857 |
| NSA2 | 2.21978832 | SMC2 | 2.17909794 | MCM7 | 2.14006145 | OGA | 2.10644258 |
| RL10 | 2.21759483 | CSTF2 | 2.17639929 | NMI | 2.13906057 | SMU1 | 2.10629676 |
| SRP14 | 2.21737187 | CDK9 | 2.17573353 | NONO | 2.13802278 | UBP8 | 2.10587225 |
| PGAM5 | 2.21520426 | GMFB | 2.17144405 | CCD47 | 2.13696638 | NPM3 | 2.10554617 |
| AN32E | 2.21422792 | ERO1A | 2.1705538 | TRXR2 | 2.13369641 | PRKRA | 2.10530188 |
| RS21 | 2.21105002 | COX41 | 2.1705235 | MTX1 | 2.12935234 | U520 | 2.10473112 |
| MSH2 | 2.21010392 | RFFL | 2.17015526 | DYHC1 | 2.12926672 | TIM9 | 2.10403967 |
| SCMC1 | 2.21006017 | ELYS | 2.16932907 | GPHRB | 2.12597357 | CUL5 | 2.10391997 |
| H3PS2 | 2.20933325 | HNRPR | 2.16809786 | BTAF1 | 2.12596091 | NP1L4 | 2.10384328 |
| RB3GP | 2.20741353 | RS6 | 2.16801157 | AN32B | 2.12497548 | IST1 | 2.10368051 |
| CUL3 | 2.20701631 | PREP | 2.16675231 | AT1B1 | 2.12432852 | SFXN1 | 2.10345195 |

| Protein | Foldchange | Protein | Foldchange | Protein | Foldchange | Protein | Foldchange |
| --- | --- | --- | --- | --- | --- | --- | --- |
| CEBPZ | 2.10314371 | RL5 | 2.06717251 | SON | 2.04316606 | KC1A | 2.00468918 |
| FUMH | 2.10249553 | RBM34 | 2.06659243 | IMA3 | 2.04175669 | S23IP | 2.00405241 |
| SYJ2B | 2.10150047 | PUR8 | 2.06652434 | CSN4 | 2.04034194 | PCM1 | 2.00332317 |
| PSMD4 | 2.10126459 | RBM4 | 2.06609428 | ESYT1 | 2.03925083 | DDB1 | 2.0028384 |
| SNW1 | 2.09905285 | ABHEB | 2.0654589 | EXOS7 | 2.03847305 | COX5B | 2.00257023 |
| STMN1 | 2.0982114 | GFPT1 | 2.06510619 | SUCB1 | 2.0382212 | RPN2 | 2.00189215 |
| ABRAL | 2.09774134 | PGAM1 | 2.06463665 | RABE2 | 2.0372737 | PSME2 | 2.00147761 |
| IMPA3 | 2.0972929 | SYK | 2.06426776 | ANXA6 | 2.0365299 | KTN1 | 2.00005032 |
| TOM34 | 2.09703205 | AATC | 2.0632581 | PSB1 | 2.03628596 | AP2B1 | 1.9996314 |
| RS4X | 2.09651101 | EXOS1 | 2.06297796 | PFKAM | 2.03604194 | LYRM7 | 1.99961158 |
| SYWC | 2.09623238 | CKAP4 | 2.06263896 | KGUA | 2.03486037 | CC124 | 1.99846343 |
| DHB4 | 2.09151633 | NUP62 | 2.06215572 | TBL3 | 2.03424219 | ARMC9 | 1.99790268 |
| PRS8 | 2.09025707 | TCPE | 2.0610143 | TOLIP | 2.03290578 | RS3 | 1.99778338 |
| RADI | 2.09013251 | IPO8 | 2.05929371 | VPS29 | 2.0300561 | SYAC | 1.99709942 |
| FAH2A | 2.08782334 | VPS35 | 2.05830598 | PCAT1 | 2.02846472 | DDX17 | 1.99532451 |
| FAF2 | 2.08683633 | ATPB | 2.05797919 | CATA | 2.02826128 | RAN | 1.99448808 |
| SNAA | 2.0850365 | AP3D1 | 2.05762114 | GARS | 2.02692639 | RPC3 | 1.99441624 |
| PPCE | 2.08500928 | DPP3 | 2.05752561 | ECHD1 | 2.02466664 | FTO | 1.99381483 |
| BID | 2.08369317 | NOC2L | 2.05750932 | NU155 | 2.02442961 | IMDH1 | 1.99234583 |
| EXOS2 | 2.08254717 | NAKD2 | 2.05737131 | THIC | 2.02301908 | GBRL2 | 1.99086204 |
| UCRI | 2.08238655 | TOM70 | 2.05686972 | PPME1 | 2.02149454 | RS7 | 1.9898694 |
| CPIN1 | 2.0816516 | S39AE | 2.0559196 | ARF5 | 2.01631022 | RPN1 | 1.98954634 |
| DIAP1 | 2.08042849 | PPHLN | 2.05431127 | RL26L | 2.01611773 | CD59 | 1.98919197 |
| COX20 | 2.08006639 | PRS4 | 2.05311131 | RSMN | 2.0157341 | EIF3H | 1.98894952 |
| STAM1 | 2.07959496 | PLSI | 2.05280282 | VP33A | 2.01547963 | FUBP2 | 1.98733328 |
| RBM25 | 2.07925998 | VTI1B | 2.05215366 | XPO1 | 2.01540311 | RU2A | 1.98612498 |
| MTAP | 2.07925076 | THIM | 2.05078853 | EIF1A | 2.01539988 | TYB4 | 1.98597426 |
| TLE5 | 2.0784268 | CMBL | 2.050077 | RFC5 | 2.01527863 | DNJA2 | 1.98571118 |
| AT2B1 | 2.07813321 | CROCC | 2.04983493 | DECR | 2.01305358 | ATPG | 1.98568268 |
| HMGN2 | 2.07615853 | MRM3 | 2.0491614 | KEAP1 | 2.01226577 | PCNA | 1.98388578 |
| PYGB | 2.07584983 | RPAC2 | 2.04890034 | AR6P1 | 2.01179034 | PFD4 | 1.98385164 |
| DHPR | 2.07556985 | TMEDA | 2.04831838 | PRPF3 | 2.01160195 | TCPZ | 1.98361428 |
| SWP70 | 2.07371917 | CE170 | 2.04795066 | ROMO1 | 2.01149371 | P53 | 1.98327537 |
| TFP11 | 2.07369611 | B2MG | 2.04613676 | UBP5 | 2.01020198 | PAAF1 | 1.98308925 |
| BTF3 | 2.07244057 | PEBB | 2.04613674 | RS14 | 2.00989932 | FMC1 | 1.98279537 |
| MLP3B | 2.07200542 | RAE1L | 2.04583635 | SODM | 2.00907552 | ACOT1 | 1.98232073 |
| ATG9A | 2.07186646 | CD166 | 2.04394514 | TCPH | 2.00882136 | PSIP1 | 1.98212284 |
| ATLA2 | 2.07146142 | DYLT1 | 2.04372459 | G6PD | 2.0083905 | PPIB | 1.98131252 |
| AL7A1 | 2.07087068 | CDC5L | 2.043455 | DPM1 | 2.00506385 | EMC7 | 1.98047379 |

| Protein | Foldchange | Protein | Foldchange | Protein | Foldchange | Protein | Foldchange |
| --- | --- | --- | --- | --- | --- | --- | --- |
| RS13 | 1.97973429 | FAKD4 | 1.95798746 | MPPA | 1.93440958 | XPO7 | 1.91246879 |
| PRDX2 | 1.97865297 | GSHB | 1.9579653 | EIF3D | 1.93377863 | RBM39 | 1.91071616 |
| EXOS4 | 1.97855084 | RTCB | 1.95748663 | RL9 | 1.93284755 | KCC2G | 1.91067025 |
| DFFA | 1.97795854 | H90B2 | 1.95742595 | PNKP | 1.93177108 | LMNA | 1.90975663 |
| PAF1 | 1.97766235 | SRP09 | 1.95435801 | CLCA | 1.9315042 | CISY | 1.90938237 |
| NOC4L | 1.97749716 | PURA | 1.95329059 | S61A1 | 1.93131019 | FBX22 | 1.90916051 |
| AGK | 1.97727638 | PP1R8 | 1.95237954 | ILK | 1.93103448 | ACOC | 1.90863492 |
| PARP1 | 1.97667638 | RL23 | 1.95235261 | ERLN2 | 1.9307483 | UB2R2 | 1.90781582 |
| SERC | 1.97617137 | 8ODP | 1.95200344 | EIF3L | 1.92952122 | HNRH1 | 1.90762818 |
| DHE3 | 1.97604915 | C1TC | 1.95146307 | AP1S1 | 1.92948254 | MYH9 | 1.90555375 |
| NSMA | 1.97523786 | RL7L | 1.95081334 | SYQ | 1.92834541 | FAD1 | 1.90422716 |
| EIF1 | 1.97419928 | IDI1 | 1.95043169 | RO60 | 1.9278298 | IF4A1 | 1.90411667 |
| IGBP1 | 1.97358207 | ITAV | 1.95007977 | SMC4 | 1.92657933 | PUR9 | 1.90373277 |
| PSMG3 | 1.97254613 | TTL12 | 1.95003578 | FIS1 | 1.92594136 | QCR2 | 1.902685 |
| RAB6A | 1.97228853 | VTA1 | 1.94910612 | MIC60 | 1.92589688 | RBM10 | 1.90084067 |
| MRT4 | 1.9712048 | GOLM1 | 1.94905194 | SC24C | 1.92512248 | SNUT1 | 1.90083131 |
| NCBP1 | 1.97098211 | C560 | 1.94890684 | PSDE | 1.92478659 | S10AA | 1.89919566 |
| PRAF3 | 1.97081122 | XPP1 | 1.9480009 | KPYM | 1.92436586 | BASI | 1.89897474 |
| R39L5 | 1.97066717 | SSF1 | 1.9477199 | LAP2B | 1.9238434 | EFTU | 1.898406 |
| SRP54 | 1.97020988 | LKHA4 | 1.94749577 | U5S1 | 1.92183243 | NPM | 1.89838772 |
| GBB1 | 1.96927891 | RL10A | 1.94723971 | TOP2B | 1.9206499 | DSRAD | 1.89807407 |
| VDAC2 | 1.96901247 | SERB | 1.94707016 | CEP43 | 1.92010083 | GLGB | 1.89794254 |
| RAB18 | 1.96863358 | NDUS1 | 1.94666763 | LSM4 | 1.9199235 | SYIC | 1.89767359 |
| AT131 | 1.96856149 | IWS1 | 1.94425727 | GSTO1 | 1.91971989 | REEP5 | 1.89750441 |
| ACTN1 | 1.96806667 | TCPB | 1.94386893 | TXND5 | 1.9179672 | DRG1 | 1.89747303 |
| ARF4 | 1.96702815 | LGUL | 1.94366481 | CLPP | 1.91776809 | NUP43 | 1.8973944 |
| MGDP1 | 1.96635588 | RAB5C | 1.94306948 | HNRPC | 1.91740917 | ATX10 | 1.89693085 |
| PAR6B | 1.96473459 | NAMPT | 1.94289192 | LMAN2 | 1.91739659 | RL15 | 1.89598821 |
| LRC47 | 1.96330483 | RIC8A | 1.94285647 | AL9A1 | 1.91736448 | NFH | 1.89539674 |
| GRSF1 | 1.96286223 | DEST | 1.94281743 | 4F2 | 1.91730793 | NDUBA | 1.89536466 |
| SC31A | 1.96123718 | SF3B6 | 1.94210937 | LSM12 | 1.91702138 | PA1B2 | 1.89523525 |
| EIF2D | 1.96095894 | RAGP1 | 1.93953625 | LMAN1 | 1.91659724 | NACC1 | 1.89499532 |
| SEPT9 | 1.96025431 | NPL4 | 1.93925955 | CSN1 | 1.9158498 | RM49 | 1.89480786 |
| AK1A1 | 1.95955665 | PPID | 1.93875193 | UBR4 | 1.91460046 | COR1C | 1.89463107 |
| STAT1 | 1.95943318 | PABP2 | 1.93727938 | DECR2 | 1.91427677 | CCN1 | 1.89432887 |
| SPP2A | 1.95938885 | MYL6 | 1.93704336 | VPS4A | 1.91408627 | GIPC1 | 1.89320296 |
| SYRC | 1.95909162 | NOMO1 | 1.93669809 | BIEA | 1.91370017 | LARP7 | 1.8926506 |
| PTCD3 | 1.95871703 | USO1 | 1.93668767 | OTU6B | 1.91310491 | PSB5 | 1.89259495 |
| SPTB2 | 1.95808718 | RL13A | 1.93619636 | GMDS | 1.91249664 | RRFM | 1.89256624 |

| Protein | Foldchange | Protein | Foldchange | Protein | Foldchange | Protein | Foldchange |
| --- | --- | --- | --- | --- | --- | --- | --- |
| DHX30 | 1.89235234 | PUR1 | 1.86819205 | IDH3A | 1.84645406 | PYRG1 | 1.82201707 |
| HNRPM | 1.89220376 | RS9 | 1.86810062 | NDUA8 | 1.84632297 | UBQL4 | 1.82146093 |
| EMAL4 | 1.89201853 | P3H1 | 1.8675338 | PGK1 | 1.84593537 | ATIF1 | 1.82100137 |
| NLE1 | 1.89026983 | ABCD3 | 1.86737835 | HAT1 | 1.84560138 | PUR4 | 1.82003816 |
| GEPH | 1.89025436 | SYSC | 1.86714656 | SNUT2 | 1.84492759 | TIM50 | 1.81952991 |
| DHX9 | 1.88948221 | FUS | 1.86608553 | CSN8 | 1.84401287 | ACINU | 1.81933377 |
| TBB4B | 1.88675237 | NLTP | 1.86583835 | RL21 | 1.84334583 | UFD1 | 1.81875276 |
| MTPN | 1.88639856 | GLOD4 | 1.865346 | RNF25 | 1.8430268 | SC61B | 1.8172801 |
| HDAC1 | 1.88635582 | FUBP3 | 1.86348344 | CDK5 | 1.84245137 | RSRC2 | 1.81727284 |
| SEC13 | 1.88561633 | NB5R3 | 1.86344324 | MPRD | 1.84238136 | MCM2 | 1.81720032 |
| ACAD9 | 1.8851573 | RAC1 | 1.86333866 | AP3M1 | 1.84214686 | CLH1 | 1.81679833 |
| PGM1 | 1.88408323 | BCLF1 | 1.8631745 | GDS1 | 1.84122718 | LPPRC | 1.81612836 |
| 1433E | 1.88321719 | CFA20 | 1.86243551 | FLNB | 1.84112951 | RS12 | 1.81500194 |
| HEBP2 | 1.88308601 | TMCO1 | 1.86228407 | COTL1 | 1.84039881 | CHD4 | 1.81311999 |
| TEX10 | 1.88302038 | TYSD1 | 1.86204608 | RAB34 | 1.84034018 | COPD | 1.81296605 |
| NXP20 | 1.88271808 | F10A1 | 1.86191271 | PDCD5 | 1.83982684 | SERPH | 1.81295253 |
| UBC9 | 1.88094377 | ACTZ | 1.86016673 | GHITM | 1.83846751 | NTF2 | 1.81268297 |
| SYEP | 1.88070612 | KAD3 | 1.86002886 | ATPA | 1.83821168 | ROA3 | 1.81230495 |
| VATA | 1.87990706 | DCTN2 | 1.85883018 | BUB3 | 1.83801382 | CUL2 | 1.81214008 |
| PLIN3 | 1.87981724 | RM43 | 1.85785756 | CAND1 | 1.83736949 | SC24D | 1.81203092 |
| AATM | 1.87963537 | ETFB | 1.85715133 | NDUA5 | 1.83705624 | SRSF5 | 1.81119143 |
| CARL1 | 1.87942364 | BAP31 | 1.8567236 | PLXB2 | 1.83665463 | PRP19 | 1.81074283 |
| RM04 | 1.87895644 | SYLM | 1.85550787 | AIMP2 | 1.83582259 | GOSR1 | 1.80984854 |
| P5CR3 | 1.87861082 | ARPC2 | 1.8554925 | G3BP2 | 1.83558658 | TE2IP | 1.80982645 |
| WDR26 | 1.87734201 | ACADM | 1.85381118 | RAB6B | 1.83513882 | AAMP | 1.8089461 |
| GGCT | 1.87604133 | EMC1 | 1.85345646 | RL35A | 1.83424038 | PFD5 | 1.80835324 |
| PTPS | 1.87529632 | IF2G | 1.85330042 | UPP1 | 1.83288651 | VASP | 1.80824991 |
| HSP7E | 1.87494506 | CAZA1 | 1.85328247 | TPD54 | 1.83247685 | GDIA | 1.80796309 |
| CPNE2 | 1.87385095 | ARP2 | 1.85323639 | RRP5 | 1.8324549 | SKP1 | 1.80764302 |
| ADPPT | 1.87294048 | RAB9A | 1.85316595 | GSHR | 1.83098804 | HBS1L | 1.80645482 |
| ANXA7 | 1.87182296 | CH60 | 1.85133992 | ENOG | 1.83066034 | AHNK | 1.80561629 |
| NH2L1 | 1.87154003 | SCPDL | 1.85057146 | DCAF8 | 1.83026624 | MOGS | 1.80338609 |
| SMC3 | 1.87091528 | PRAF2 | 1.85009009 | PSMD6 | 1.82968451 | RM34 | 1.80287066 |
| HACL2 | 1.87054758 | COPB | 1.84882446 | PSB6 | 1.82585438 | DCTN1 | 1.80202671 |
| UB2L3 | 1.87040489 | SORCN | 1.84881524 | MTCH2 | 1.82550245 | RAB5B | 1.80181078 |
| CYFP1 | 1.86950998 | BOLA2 | 1.84735503 | HNRPU | 1.82421146 | PORIM | 1.80057939 |
| SF3B2 | 1.86913832 | IF2A | 1.84716971 | SYMC | 1.82384956 | IF2P | 1.79959161 |
| AMPL | 1.86869056 | 3HIDH | 1.84673252 | DIC | 1.82379765 | MCM4 | 1.79948285 |
| CCD58 | 1.86852024 | EBP | 1.84646249 | PARVA | 1.82333258 | 2A5D | 1.79932891 |

| Protein | Foldchange | Protein | Foldchange | Protein | Foldchange | Protein | Foldchange |
| --- | --- | --- | --- | --- | --- | --- | --- |
| GLRX1 | 1.79915296 | MYL9 | 1.77245892 | NC2A | 1.74986162 | PAIP1 | 1.7322656 |
| SC16A | 1.79570892 | GRHPR | 1.77149817 | ANX11 | 1.749633 | PUR2 | 1.73219979 |
| P66B | 1.79466723 | GCN1 | 1.77036274 | WDR43 | 1.7489826 | BAF | 1.73123864 |
| WDHD1 | 1.79385923 | KAD1 | 1.77030902 | PRKDC | 1.74879064 | CTND1 | 1.73113396 |
| GILT | 1.79293361 | MDHM | 1.77012643 | S10AD | 1.74876921 | BIP | 1.73067163 |
| SMN | 1.79291919 | PRS10 | 1.76772028 | SCRN1 | 1.74866579 | HMGB1 | 1.73044596 |
| RAB7A | 1.79285998 | ATPO | 1.76748307 | KHDR1 | 1.74864346 | TXLNA | 1.73006805 |
| TBB5 | 1.79210821 | PP6R2 | 1.76639492 | UBA3 | 1.74845961 | RB27A | 1.72892009 |
| EWS | 1.79160518 | UBA1 | 1.76613983 | TBA1A | 1.74754626 | NCOA5 | 1.72864287 |
| ITB1 | 1.79095038 | CLU | 1.76491743 | SSRP1 | 1.74740721 | CLPT1 | 1.72800133 |
| CYTB | 1.79092274 | SYYC | 1.7648472 | SAP | 1.74500258 | HMGA2 | 1.72702201 |
| KLC1 | 1.78976918 | GPN1 | 1.764714 | NMT1 | 1.74429151 | KATL2 | 1.72613209 |
| PLEC | 1.78953108 | BCKD | 1.76469605 | GSK3A | 1.7440056 | ARP3 | 1.72588735 |
| NASP | 1.78909273 | EIF3E | 1.76291887 | PSMD1 | 1.74310208 | NOL11 | 1.7258537 |
| TSN | 1.78835979 | PSMD3 | 1.7616958 | ATAD1 | 1.74295319 | RTF1 | 1.72551645 |
| EXOS6 | 1.7876693 | CCDB1 | 1.76157737 | MOES | 1.74280797 | TM9S4 | 1.72489796 |
| SF3A1 | 1.78649981 | PUR6 | 1.76117464 | LDHA | 1.74279487 | ANXA5 | 1.72487407 |
| TMED2 | 1.7863899 | 6PGD | 1.76100499 | TERA | 1.74277343 | H2B1L | 1.72381005 |
| LMNB1 | 1.78634382 | HEXI1 | 1.76084958 | NQO2 | 1.74245872 | DP13A | 1.72351785 |
| ANXA2 | 1.78572217 | STRAP | 1.76072797 | ERF3A | 1.74180748 | RU2B | 1.72143429 |
| THUM1 | 1.78429699 | ML12A | 1.75966625 | P66A | 1.74178918 | 1433F | 1.72104258 |
| DNJC8 | 1.7838656 | PRP8 | 1.75860212 | ABCF1 | 1.73990264 | RS18 | 1.72101269 |
| NDKB | 1.78217129 | CNN3 | 1.7575574 | TRIPC | 1.73980564 | DDX3X | 1.72074722 |
| RL4 | 1.78066642 | CPNE3 | 1.75716961 | RHOC | 1.73955174 | GWL | 1.72048469 |
| SGPL1 | 1.77996965 | SAMH1 | 1.75687473 | EXOSX | 1.73913043 | H2AY | 1.72022662 |
| RS15A | 1.77978967 | PRDX4 | 1.75684682 | ROCK2 | 1.73867878 | ENPL | 1.71945936 |
| RM20 | 1.77973312 | TOPK | 1.7568113 | AIP | 1.73809876 | RRP1 | 1.71828098 |
| RCC2 | 1.77910412 | CCHL | 1.75520743 | CSN5 | 1.73786408 | ELP2 | 1.71800351 |
| DHSO | 1.77842814 | RAB5A | 1.75482854 | MTX2 | 1.73775736 | CX038 | 1.71699609 |
| IF2B1 | 1.77806918 | GLCM | 1.75482441 | MTREX | 1.73670536 | UBE2T | 1.71685475 |
| NFS1 | 1.77730901 | RFC4 | 1.75377082 | APOL2 | 1.73642447 | ARPC3 | 1.71650408 |
| STML2 | 1.77554049 | PDLI5 | 1.75310458 | CCAR2 | 1.73612591 | VATC1 | 1.71527778 |
| EIF3K | 1.7754345 | UBC12 | 1.75306379 | TCTP | 1.7356074 | HIBCH | 1.71352037 |
| FKBP2 | 1.77496501 | SNF8 | 1.75298819 | MIC19 | 1.73545305 | ATP6 | 1.713279 |
| TBCA | 1.77483363 | TBB2A | 1.75209719 | PLOD1 | 1.73396913 | NELFE | 1.71262255 |
| CL029 | 1.77449807 | HCFC1 | 1.7519449 | TCPG | 1.73391673 | PSA6 | 1.71186937 |
| PCBP1 | 1.7744263 | IPO5 | 1.75167487 | EF1G | 1.73356495 | TBL1R | 1.71124018 |
| PCKGM | 1.77389401 | PTBP1 | 1.75050832 | ENOA | 1.73346056 | TM165 | 1.71098037 |
| UTP15 | 1.77372574 | RL22L | 1.75030777 | NIPS1 | 1.73249528 | RL29 | 1.71091543 |

| Protein | Foldchange | Protein | Foldchange | Protein | Foldchange | Protein | Foldchange |
| --- | --- | --- | --- | --- | --- | --- | --- |
| THIO | 1.7101122 | RALA | 1.68951555 | CSK21 | 1.6710002 | HNRPL | 1.64886061 |
| PFD1 | 1.70929342 | TCEA1 | 1.68798073 | BCCIP | 1.67010735 | CPSF6 | 1.64776885 |
| ZFAN6 | 1.70872606 | IPYR | 1.68756134 | STAM2 | 1.66913472 | MAP11 | 1.64731198 |
| PTPA | 1.70858594 | VAPA | 1.68736333 | ANM1 | 1.66823469 | RS26 | 1.6466721 |
| RL26 | 1.70837359 | ETFA | 1.68722412 | TCPA | 1.66796728 | P3H3 | 1.64599847 |
| MATR3 | 1.70816812 | CD11B | 1.68622536 | EI2BB | 1.6676021 | PSMD9 | 1.645686 |
| CASP6 | 1.70784656 | WDR1 | 1.68490058 | VIGLN | 1.66700405 | UBP14 | 1.64493886 |
| RM24 | 1.70737812 | PELP1 | 1.68486224 | DEK | 1.66585996 | ADAS | 1.64493781 |
| SH3L1 | 1.70609331 | BT3L4 | 1.68485895 | HINT1 | 1.66510743 | UB2V1 | 1.6432319 |
| SUN1 | 1.70551013 | NHRF1 | 1.68352288 | ARF1 | 1.66418032 | CAPZB | 1.64288237 |
| DDAH1 | 1.70500826 | WDR82 | 1.68344021 | AP2M1 | 1.66402772 | YBOX1 | 1.64264324 |
| ARFG3 | 1.70485368 | SF3B1 | 1.68302599 | H32 | 1.66360963 | TES | 1.64251259 |
| NOG1 | 1.70319016 | DNJA1 | 1.682501 | EIF3C | 1.66309503 | LIS1 | 1.64247461 |
| GTF2I | 1.70296721 | PSA4 | 1.68169067 | MEA1 | 1.66283748 | UBP47 | 1.64208061 |
| SAR1B | 1.70252637 | 1433S | 1.68151213 | WDR18 | 1.66279044 | RL35 | 1.64204046 |
| CYRIB | 1.70232448 | TCRG1 | 1.68083149 | 1433Z | 1.66185906 | GNA1 | 1.64191627 |
| XPO5 | 1.70208569 | UB2V2 | 1.68062544 | LAP2A | 1.66180669 | TMM33 | 1.64121079 |
| ACL6A | 1.70130144 | RS24 | 1.68059418 | MTA1 | 1.66103841 | VA0D1 | 1.6405158 |
| SUMF2 | 1.70012262 | ZN638 | 1.680338 | SNAG | 1.65981822 | DIM1 | 1.63986471 |
| GBP1 | 1.69983878 | ENOPH | 1.680245 | HNRPD | 1.65793181 | KAD2 | 1.63924169 |
| COPA | 1.69958019 | PLST | 1.68021456 | ERH | 1.65734783 | AKP8L | 1.63882651 |
| FPPS | 1.69918812 | LSR | 1.67993844 | IMB1 | 1.65728795 | DDX23 | 1.63832062 |
| PDIA1 | 1.69912533 | REEP4 | 1.67908999 | RPIA | 1.65639222 | DX39A | 1.63798151 |
| PRS7 | 1.69872972 | IN35 | 1.67899639 | ORN | 1.65590288 | PESC | 1.63721941 |
| SND1 | 1.6983371 | HELLS | 1.67892189 | LAMP2 | 1.65461925 | RM15 | 1.63682137 |
| FAS | 1.6979507 | RS15 | 1.67861077 | PSB4 | 1.65447524 | CSTF3 | 1.63661853 |
| TMED9 | 1.69696742 | PSA1 | 1.67829441 | SPS1 | 1.65413534 | GLO2 | 1.63556229 |
| GSTP1 | 1.69569168 | CD2B2 | 1.6779179 | TNPO1 | 1.65394335 | OFUT1 | 1.63503589 |
| SYVC | 1.69519182 | RL22 | 1.67780789 | EFHD2 | 1.65371798 | FLNA | 1.63499875 |
| M2OM | 1.69514617 | KAP0 | 1.67776534 | MP2K1 | 1.65281434 | FLOT2 | 1.63487118 |
| PELO | 1.69443142 | ANCHR | 1.67697668 | PPCS | 1.65246852 | NOP58 | 1.63436622 |
| MAK16 | 1.69372897 | MK01 | 1.67483511 | SNX9 | 1.65210066 | RL11 | 1.63387313 |
| TXND9 | 1.69323136 | SYTC | 1.6747303 | WASF2 | 1.65140813 | SMD2 | 1.63215296 |
| KCRB | 1.69275739 | SF3B3 | 1.67434446 | G6PI | 1.65129454 | KI67 | 1.63152377 |
| CY1 | 1.69168066 | PNO1 | 1.67371485 | IF4G2 | 1.6510813 | THOC4 | 1.63119081 |
| PTGR1 | 1.69128474 | KTHY | 1.67326962 | MAPK2 | 1.6505295 | DPOD2 | 1.63047803 |
| SRSF7 | 1.69109031 | ACON | 1.67249869 | XRCC5 | 1.64944197 | DCTD | 1.62975241 |
| DC1I2 | 1.69104361 | RHOA | 1.67232849 | TAGL | 1.64940096 | IF2B2 | 1.62863261 |
| GANAB | 1.69031416 | SRC | 1.67157222 | THIOM | 1.64886137 | CALU | 1.62792109 |

| Protein | Foldchange | Protein | Foldchange | Protein | Foldchange | Protein | Foldchange |
| --- | --- | --- | --- | --- | --- | --- | --- |
| HS90A | 1.62661493 | MYO6 | 1.60825207 | RL18A | 1.58965194 | ITPA | 1.56734369 |
| ROA0 | 1.6260938 | RSSA | 1.60820963 | IDE | 1.58890838 | C1QBP | 1.5673111 |
| PP6R1 | 1.62596937 | ILEU | 1.60773967 | RBP2 | 1.58879683 | RPB2 | 1.56698193 |
| DYL1 | 1.62564748 | NSF1C | 1.6076468 | SPTN1 | 1.5880728 | RL23A | 1.56665095 |
| ACACA | 1.62519019 | CAN1 | 1.60453987 | H2AV | 1.58739123 | CAN2 | 1.56661623 |
| GLU2B | 1.62491883 | PA2G4 | 1.60447335 | GET3 | 1.5867253 | SNX6 | 1.56660811 |
| ESTD | 1.62473543 | HS71B | 1.60430522 | PGP | 1.58636454 | IDHC | 1.56497795 |
| CKAP5 | 1.62234628 | EDC3 | 1.60388311 | TKT | 1.58594458 | RT02 | 1.56226858 |
| PFD2 | 1.62152514 | CRK | 1.60386558 | NOP14 | 1.58581236 | FCL | 1.56199391 |
| EP15R | 1.61998633 | SUMO1 | 1.60323887 | TS101 | 1.58575302 | IQGA1 | 1.56149343 |
| PDE12 | 1.61929483 | EMD | 1.60296143 | RBM27 | 1.58530681 | RLP24 | 1.56086334 |
| HNRL2 | 1.61920244 | CAP1 | 1.60270608 | RL1D1 | 1.58365964 | RM53 | 1.560785 |
| NAA50 | 1.61894175 | TRIP6 | 1.60065022 | ALDOA | 1.58341451 | DC1L2 | 1.56040609 |
| HYPK | 1.61748471 | PYR1 | 1.60063274 | STAU1 | 1.58293626 | PRC1 | 1.55924449 |
| RL7 | 1.61725177 | RHOG | 1.60027084 | FEN1 | 1.58199463 | EI2BA | 1.55921593 |
| DX39B | 1.61678293 | TM160 | 1.60024882 | VDAC3 | 1.58162907 | RCN1 | 1.55879063 |
| TOIP2 | 1.61677081 | LEG1 | 1.59994903 | TM109 | 1.58142864 | EF1B | 1.55874816 |
| HMOX1 | 1.61676328 | QSOX2 | 1.59989812 | SLK | 1.58108947 | AINX | 1.55862069 |
| S35U4 | 1.61606188 | NOC3L | 1.5992878 | RL34 | 1.58061069 | USP9X | 1.55831764 |
| TCPQ | 1.61595481 | RM12 | 1.59911372 | NUP50 | 1.58046604 | PUF60 | 1.55805408 |
| TMOD3 | 1.61590318 | EF1A1 | 1.59882874 | LMNB2 | 1.58041878 | UBP7 | 1.55787092 |
| API5 | 1.61468267 | ACOT9 | 1.59869933 | RINI | 1.57989349 | B2L13 | 1.55752867 |
| AIMP1 | 1.61463057 | RPR1B | 1.59829007 | LACB2 | 1.57947757 | ETHE1 | 1.55629356 |
| COPB2 | 1.61377643 | NOL6 | 1.59787824 | COR1B | 1.57824296 | DYN2 | 1.55626958 |
| MAOM | 1.61305036 | AP1G1 | 1.59741644 | GLRX3 | 1.57805132 | CD81 | 1.55585155 |
| RL3 | 1.61302291 | RS23 | 1.59690162 | OCRL | 1.57722303 | PSD12 | 1.55559676 |
| VATG1 | 1.61271891 | L2HDH | 1.5966504 | RL37 | 1.57664122 | IF4A3 | 1.55529601 |
| IMDH2 | 1.61264379 | TPM3 | 1.5965124 | PVR | 1.57585357 | CRKL | 1.55432781 |
| PRDX3 | 1.61249519 | PPIL3 | 1.5953212 | ZO1 | 1.57543304 | PCY1A | 1.55422613 |
| GPX4 | 1.61221374 | RPAB3 | 1.5941358 | GNAI3 | 1.57477679 | QRIC1 | 1.55364471 |
| MAGD2 | 1.61207726 | IMA1 | 1.59413167 | AN32A | 1.57384712 | PDIA3 | 1.55343459 |
| MCM3 | 1.61193858 | SOAT1 | 1.59362335 | RS17 | 1.57376246 | MOT4 | 1.55272243 |
| PRDX1 | 1.6106848 | HPRT | 1.5935488 | MP2K3 | 1.57354651 | SCYL2 | 1.55244181 |
| PCBP2 | 1.61067089 | RM37 | 1.59281161 | AMRP | 1.57337566 | CBX3 | 1.55195379 |
| RS19 | 1.61023829 | STX4 | 1.59218287 | MYPT1 | 1.57306431 | ARPC5 | 1.55192585 |
| TBB3 | 1.6102277 | NIT2 | 1.5920479 | NAA10 | 1.57074292 | TCPD | 1.55147812 |
| RS3A | 1.60920542 | GNL3L | 1.59177592 | RUXF | 1.57014023 | DREB | 1.54991203 |
| NUP54 | 1.60877689 | PARK7 | 1.59160845 | RUVB1 | 1.56947005 | GRP75 | 1.54984252 |
| RBBP4 | 1.60839372 | PTN23 | 1.59104488 | MAP2 | 1.56848391 | XRCC6 | 1.54983559 |

| Protein | Foldchange | Protein | Foldchange | Protein | Foldchange | Protein | Foldchange |
| --- | --- | --- | --- | --- | --- | --- | --- |
| PAPS1 | 1.54941184 | AHSA1 | 1.52464316 | NEST | 1.50730274 | DCNL1 | 0.5813959 |
| TADBP | 1.54916072 | ILF3 | 1.52354063 | TBA1B | 1.50714056 | ADK | 0.5779149 |
| APEX1 | 1.54905142 | A16A1 | 1.52211765 | DHYS | 1.50572108 | IVD | 0.5729233 |
| GMPPA | 1.54897321 | ANM5 | 1.52153424 | XPO2 | 1.50529687 | CWC27 | 0.5674839 |
| TOIP1 | 1.54826902 | MSH6 | 1.5210407 | FKBP4 | 1.50447525 | ODPX | 0.5563151 |
| CAB45 | 1.54686688 | RB11B | 1.51946767 | DDX10 | 1.50369834 | RT16 | 0.5549528 |
| TBB8 | 1.54647368 | CHSP1 | 1.51914396 | SODC | 1.50368017 | RGS10 | 0.5543834 |
| CTGE9 | 1.54582217 | CUTA | 1.51886792 | EF2 | 1.50357221 | BRE1B | 0.550602 |
| STMN2 | 1.54557901 | HNRPK | 1.5188018 | ZN207 | 1.50343359 | ARI2 | 0.5494638 |
| APMAP | 1.54531316 | RS8 | 1.51879332 | EF1D | 1.50319227 | AGR2 | 0.5458408 |
| EXOS3 | 1.54470398 | ACLY | 1.51815595 | LDHB | 1.50316184 | IMPA1 | 0.5417025 |
| H12 | 1.54455152 | PDIA4 | 1.51797782 | HSP74 | 1.50270992 | SPCS1 | 0.5401886 |
| NDUF3 | 1.54296348 | TPP1 | 1.5176271 | HEM6 | 1.50238186 | SF3A3 | 0.5104064 |
| TPIS | 1.5428946 | RS2 | 1.51730672 | UBE2Z | 1.50095684 | PTBP3 | 0.5068042 |
| MIF | 1.54082188 | DHX29 | 1.51729625 | BYST | 1.50060484 | STT3A | 0.4938998 |
| CHTOP | 1.54071156 | KAP2 | 1.51697099 | H1X | 0.6673238 | LBR | 0.4921629 |
| PDS5A | 1.54058082 | TIPRL | 1.51694131 | SNP23 | 0.6667813 | CSK | 0.489833 |
| RS5 | 1.53926225 | DDX42 | 1.51645809 | GALT2 | 0.6617911 | DHCR7 | 0.4873254 |
| VAPB | 1.53883655 | SF3A2 | 1.51550415 | DPM3 | 0.6603193 | NFKB1 | 0.4771826 |
| PABP1 | 1.53864155 | P4HA1 | 1.51466044 | TRIR | 0.6586517 | SAAL1 | 0.4672773 |
| NUDT9 | 1.53804934 | SRP72 | 1.51444276 | UBE2O | 0.65578 | NDUS5 | 0.4592383 |
| RTN4 | 1.53794207 | TM14C | 1.5142573 | SAHH2 | 0.652857 | LAMC2 | 0.4334987 |
| RFC3 | 1.53762706 | PPIA | 1.51399891 | JAGN1 | 0.6502101 | ORC4 | 0.4270413 |
| SC23A | 1.53752685 | RTF2 | 1.51360415 | CPSF7 | 0.6482056 | ZN326 | 0.421837 |
| 1433B | 1.53708423 | DC1L1 | 1.51263095 | SYSM | 0.6368833 | U3IP2 | 0.4191759 |
| CTBP2 | 1.53670755 | HLAC | 1.51244473 | CK5P3 | 0.6338544 | ASH2L | 0.418848 |
| HLAB | 1.53553653 | KIF2C | 1.51227283 | CHP1 | 0.6337919 | YMEL1 | 0.4138812 |
| EIF3F | 1.5352587 | RUXG | 1.51204795 | NU214 | 0.6337772 | SPAG5 | 0.409654 |
| AKTS1 | 1.53292059 | NDUA6 | 1.51135195 | RN114 | 0.6326546 | HIG1A | 0.4084549 |
| RIR1 | 1.53253119 | GBB2 | 1.51120867 | RAB14 | 0.6290013 | GLYC | 0.4005538 |
| SRSF6 | 1.53238242 | TNR6 | 1.51089999 | NU160 | 0.6262947 | PP4R2 | 0.4004453 |
| ULA1 | 1.53184985 | DHRS7 | 1.51054932 | DMAC1 | 0.6255769 | PUS7 | 0.3975076 |
| RHEB | 1.53151141 | GAPD1 | 1.51020729 | S30BP | 0.609467 | WDFY1 | 0.3940464 |
| RS29 | 1.53131441 | HINT2 | 1.50996277 | GOGA2 | 0.6079159 | NADAP | 0.3905879 |
| VINC | 1.53100299 | RUVB2 | 1.50838164 | NIPA | 0.606727 | RMD1 | 0.3900915 |
| SSRG | 1.53055227 | WBP11 | 1.5079237 | GPKOW | 0.6028182 | RIDA | 0.3894816 |
| EZRI | 1.53004292 | RL14 | 1.50757353 | CCDC6 | 0.597872 | BMI1 | 0.3725404 |
| AFG32 | 1.52999967 | RD23B | 1.50737652 | TACO1 | 0.5974081 | LAMP1 | 0.3702171 |
| DDX5 | 1.52891576 | AT1B3 | 1.50736821 | NDUAC | 0.5907898 | FANCI | 0.3592238 |

| Protein | Foldchange | Protein | Foldchange | Protein | Foldchange | Protein | Foldchange |
| --- | --- | --- | --- | --- | --- | --- | --- |
| FNTA | 0.3529123 |  |  |  |  |  |  |
| ARF6 | 0.3470811 |  |  |  |  |  |  |
| NUP85 | 0.3434224 |  |  |  |  |  |  |
| PDLI3 | 0.3225777 |  |  |  |  |  |  |
| YTHD2 | 0.3185096 |  |  |  |  |  |  |
| SHLB2 | 0.3082853 |  |  |  |  |  |  |
| HMGN1 | 0.3016604 |  |  |  |  |  |  |
| DCNL5 | 0.297509 |  |  |  |  |  |  |
| K1C18 | 0.2914339 |  |  |  |  |  |  |
| SMAP1 | 0.2818747 |  |  |  |  |  |  |
| ERF3B | 0.2616573 |  |  |  |  |  |  |
| CD123 | 0.2546713 |  |  |  |  |  |  |
| LANC1 | 0.2335293 |  |  |  |  |  |  |
| NDE1 | 0.2289838 |  |  |  |  |  |  |
| MARK2 | 0.175613 |  |  |  |  |  |  |
| DCPS | 0.1473924 |  |  |  |  |  |  |
| RBBP6 | 0.1328737 |  |  |  |  |  |  |
|  |  |  |  |  |  |  |  |
|  |  |  |  |  |  |  |  |
|  |  |  |  |  |  |  |  |
|  |  |  |  |  |  |  |  |
|  |  |  |  |  |  |  |  |
|  |  |  |  |  |  |  |  |
|  |  |  |  |  |  |  |  |
|  |  |  |  |  |  |  |  |
|  |  |  |  |  |  |  |  |
|  |  |  |  |  |  |  |  |
|  |  |  |  |  |  |  |  |
|  |  |  |  |  |  |  |  |
|  |  |  |  |  |  |  |  |
|  |  |  |  |  |  |  |  |
|  |  |  |  |  |  |  |  |
|  |  |  |  |  |  |  |  |
|  |  |  |  |  |  |  |  |
|  |  |  |  |  |  |  |  |
|  |  |  |  |  |  |  |  |
|  |  |  |  |  |  |  |  |
|  |  |  |  |  |  |  |  |
|  |  |  |  |  |  |  |  |
